# Supplementary figures and images for: Directed evolution of the rRNA methylating enzyme Cfr reveals molecular basis of antibiotic resistance
Source: eLife. 2022 Jan 11;11:e70017. doi: 10.7554/eLife.70017 (PMC8752094; doi:10.7554/eLife.70017)

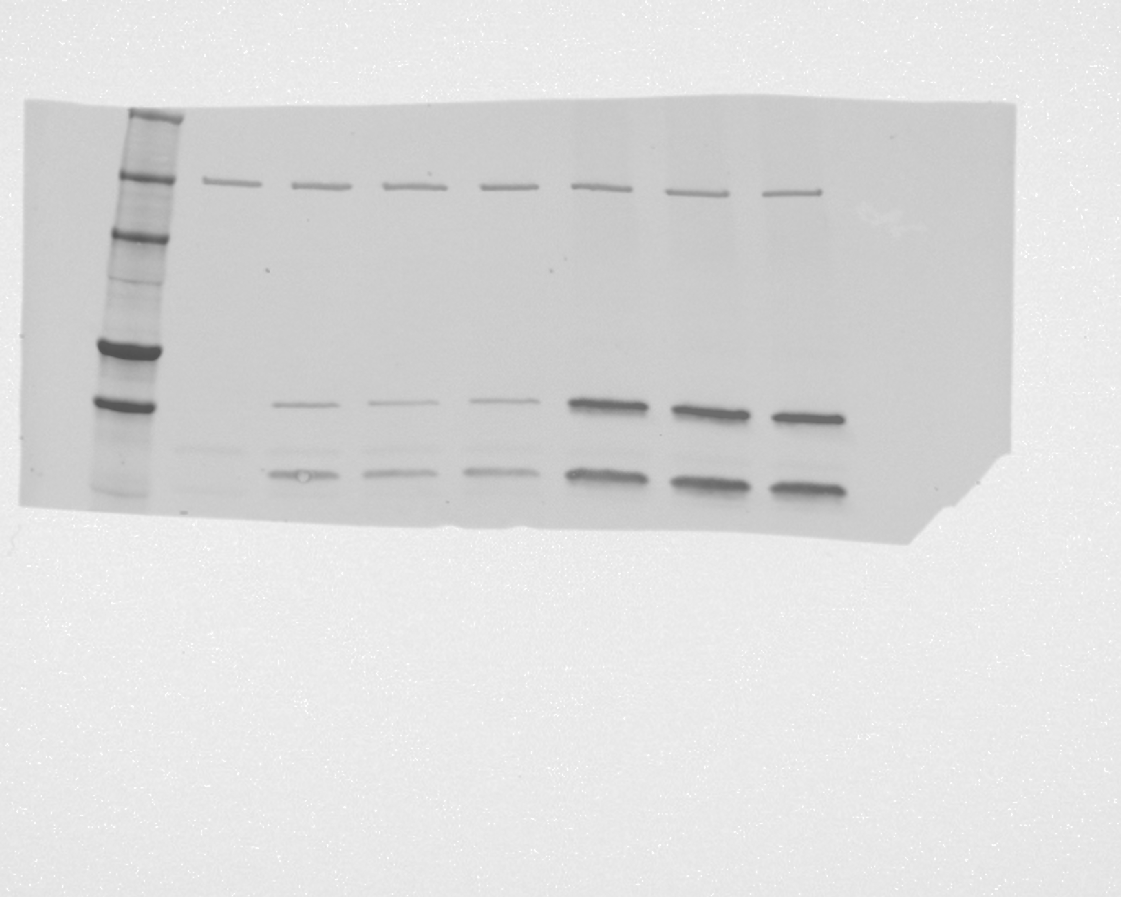

Supplement: Figure 1—source data 2. [file elife-70017-fig1-data2.zip › Figure 1 - Source Data 2 - figure supplement 1/CfrV7 no AHT (Composite).tif]

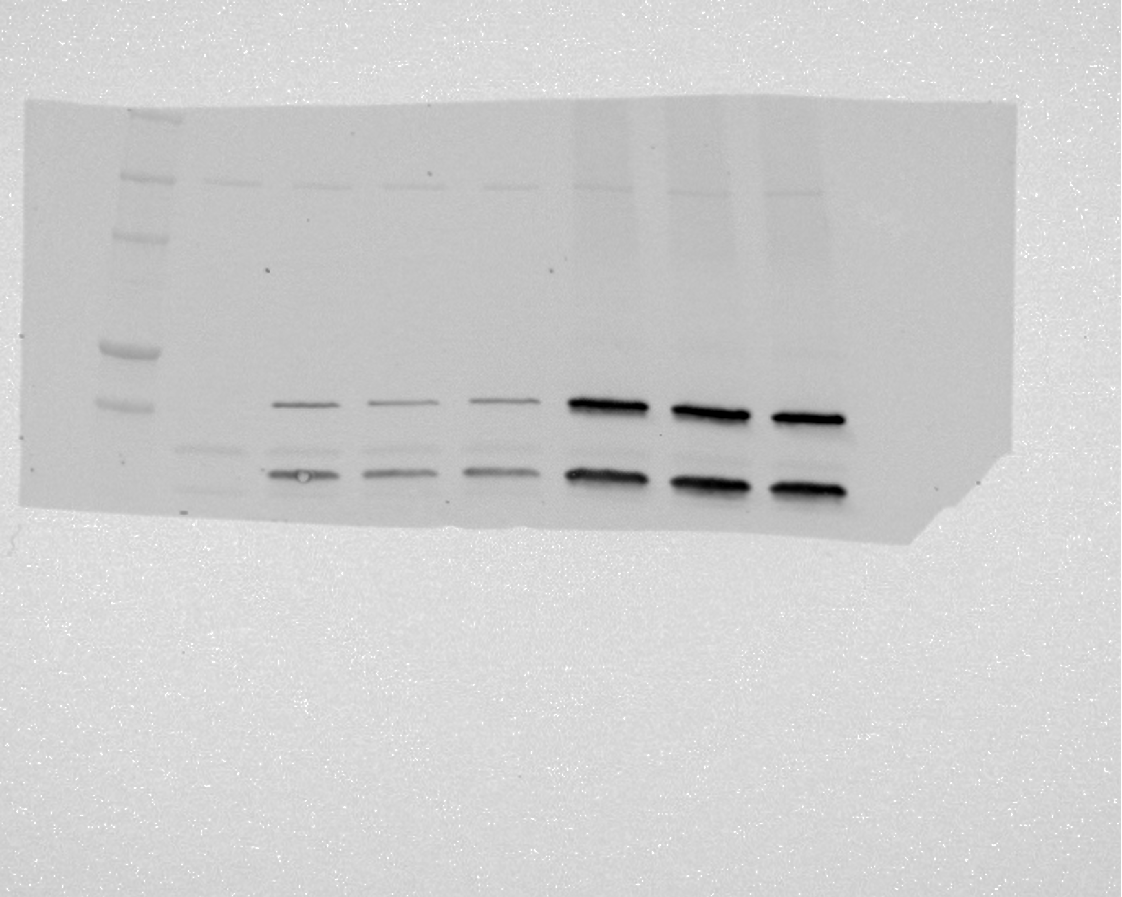

Supplement: Figure 1—source data 2. [file elife-70017-fig1-data2.zip › Figure 1 - Source Data 2 - figure supplement 1/CfrV7 no AHT (IRDye 800CW).tif]

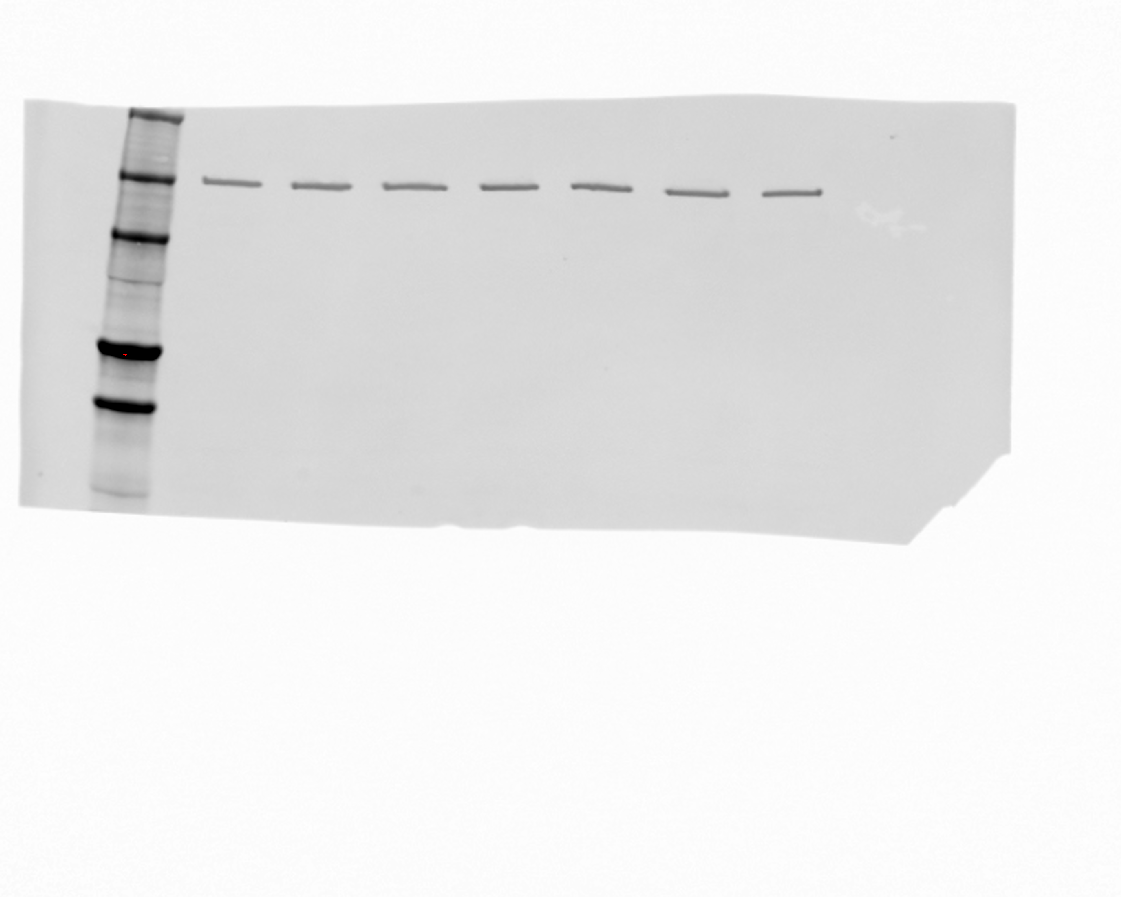

Supplement: Figure 1—source data 2. [file elife-70017-fig1-data2.zip › Figure 1 - Source Data 2 - figure supplement 1/CfrV7 no AHT (DyLight 680).tif]

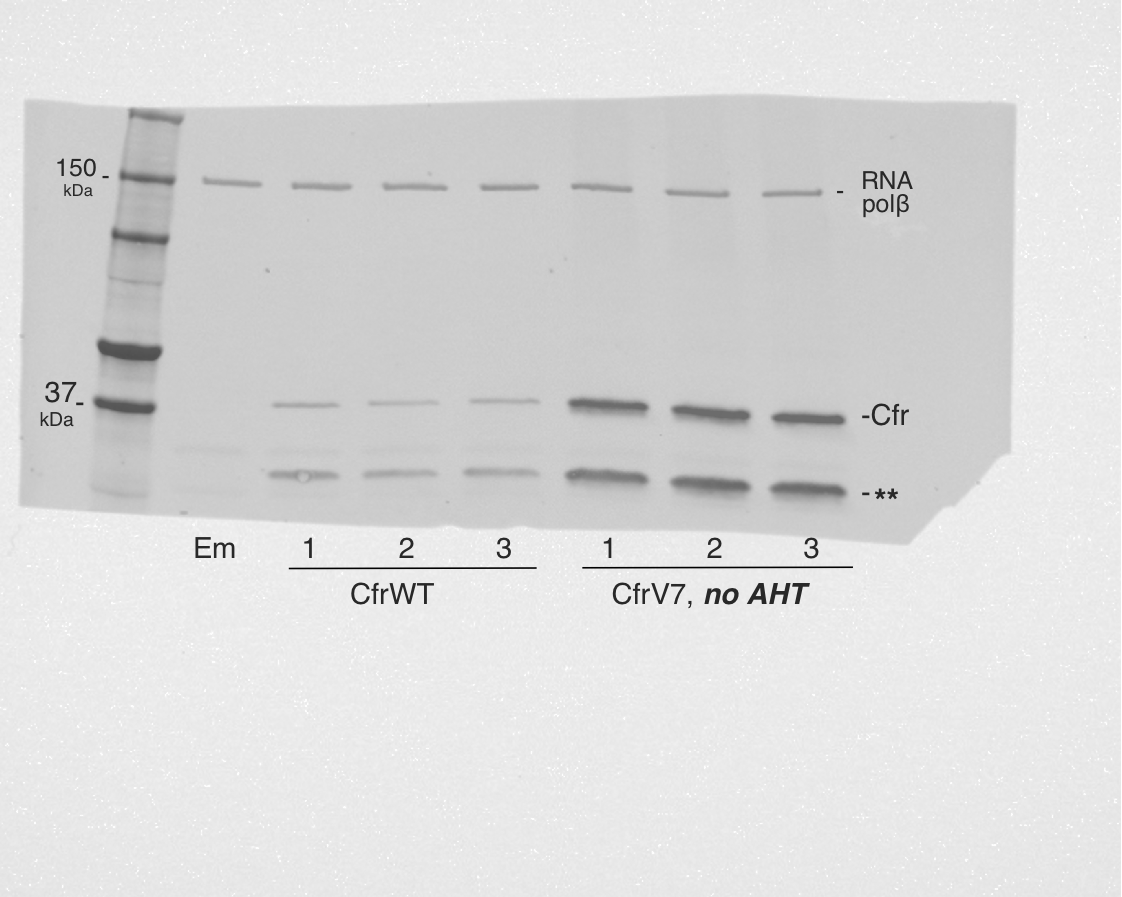

Supplement: Figure 1—source data 2. [file elife-70017-fig1-data2.zip › Figure 1 - Source Data 2 - figure supplement 1/CfrV7 no AHT (Labels).tiff]

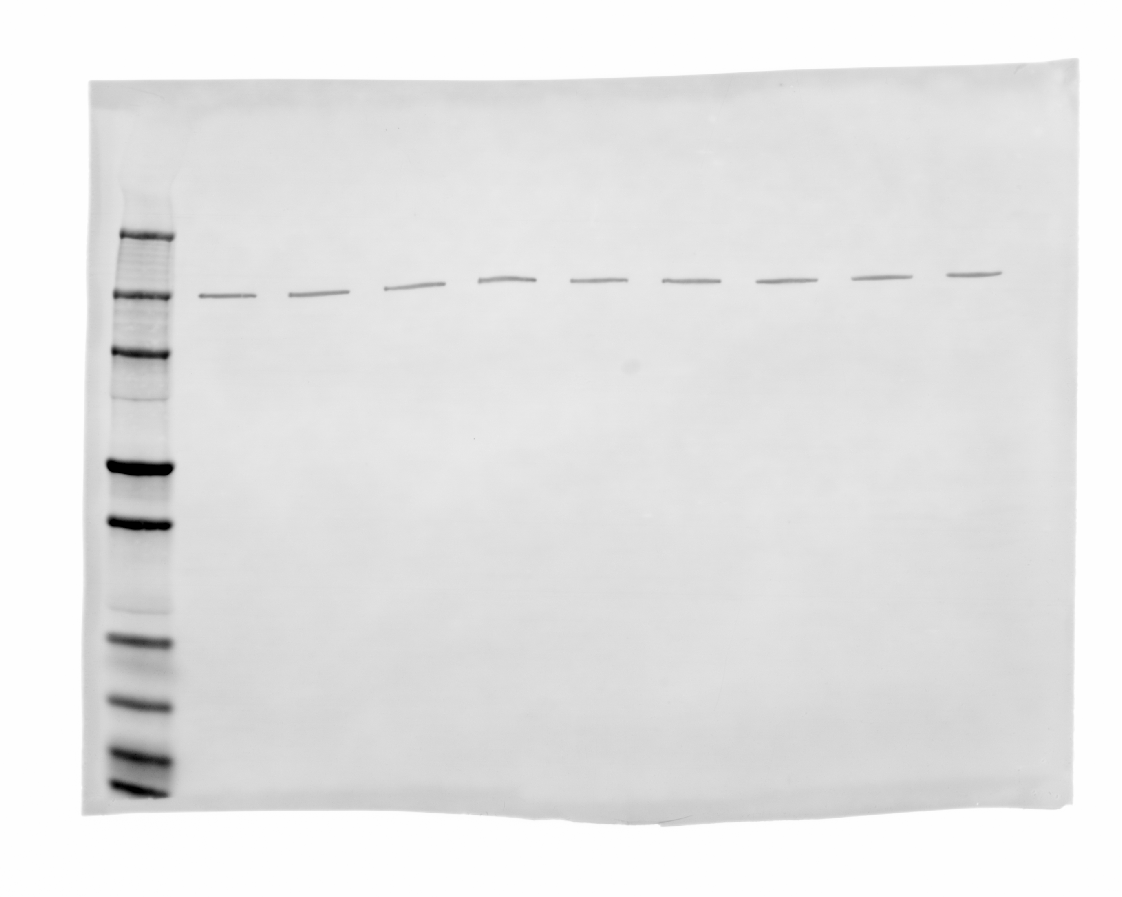

Supplement: Figure 2—source data 1. [file elife-70017-fig2-data1.zip › Figure 2 - Source Data 1/Variants (DyLight 680).tif]

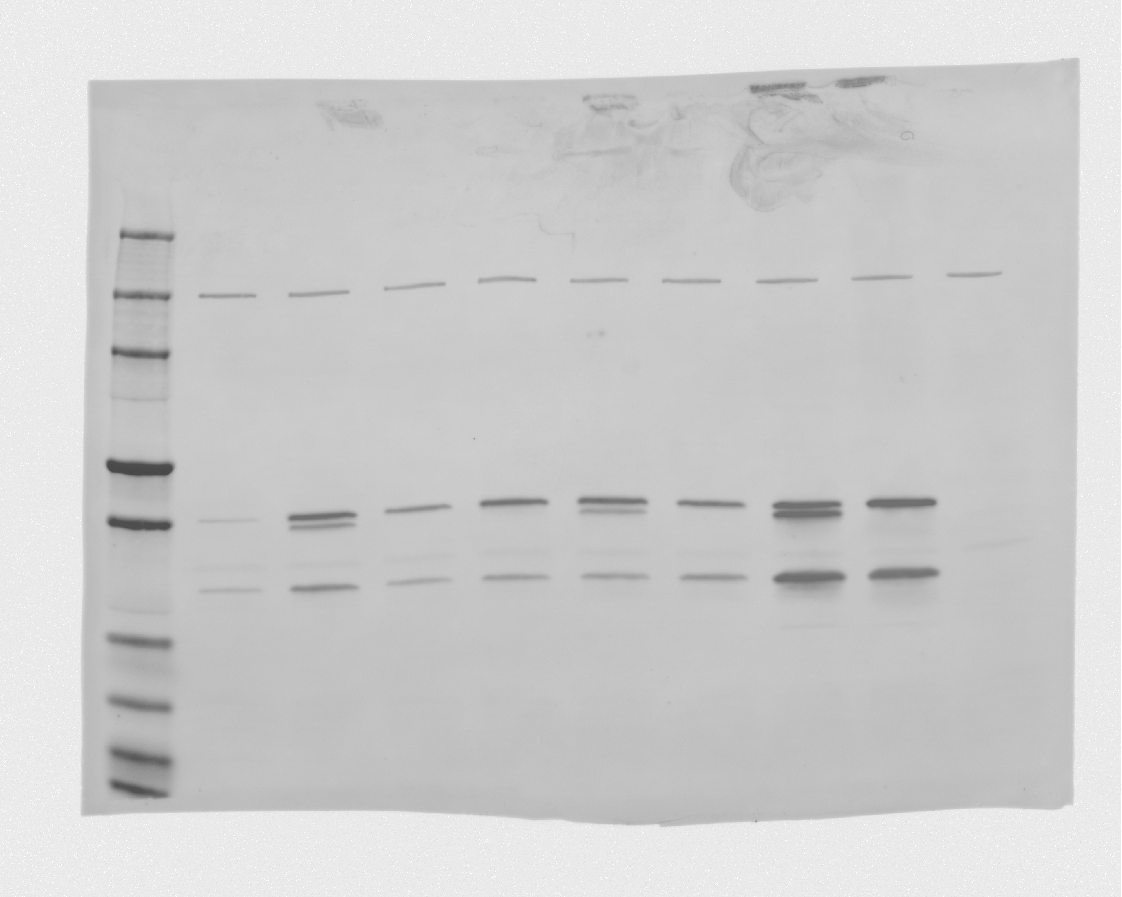

Supplement: Figure 2—source data 1. [file elife-70017-fig2-data1.zip › Figure 2 - Source Data 1/Variants (Composite).tif]

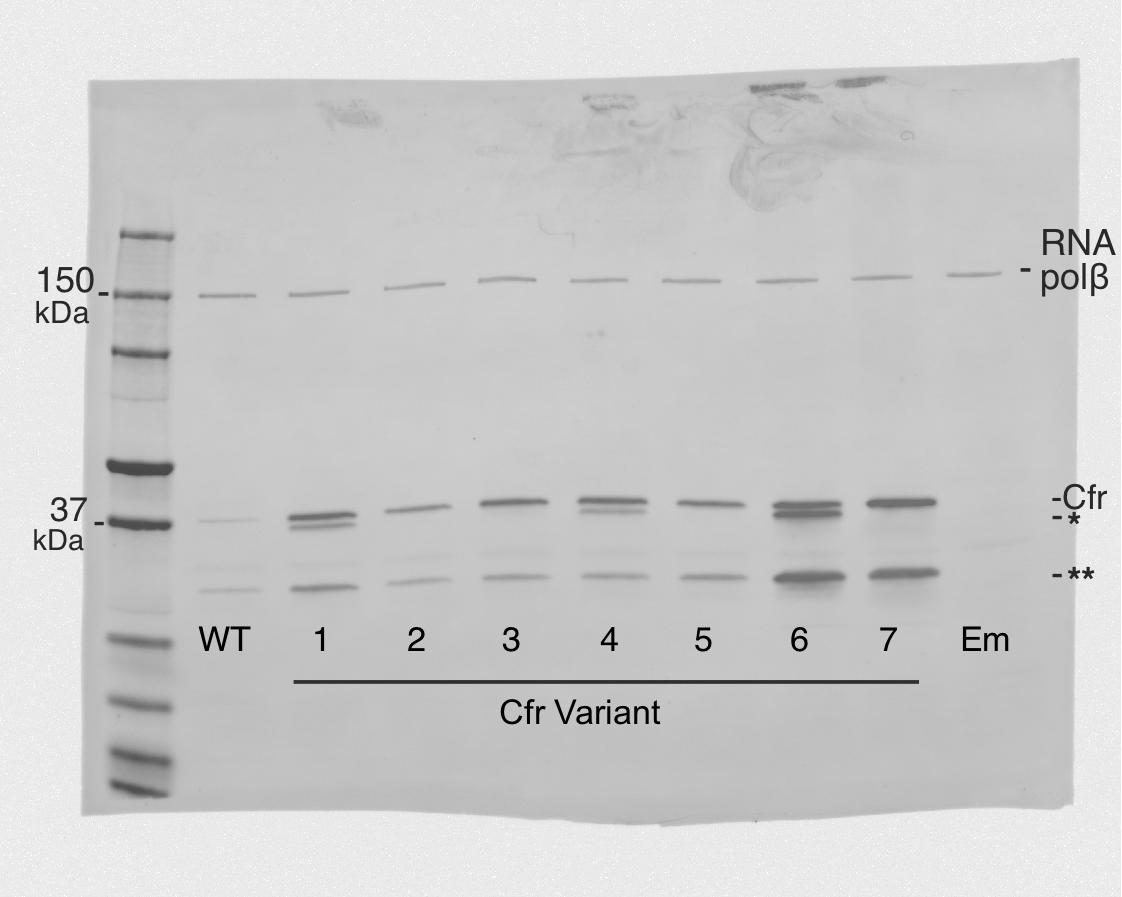

Supplement: Figure 2—source data 1. [file elife-70017-fig2-data1.zip › Figure 2 - Source Data 1/Variants (Labels).tiff]

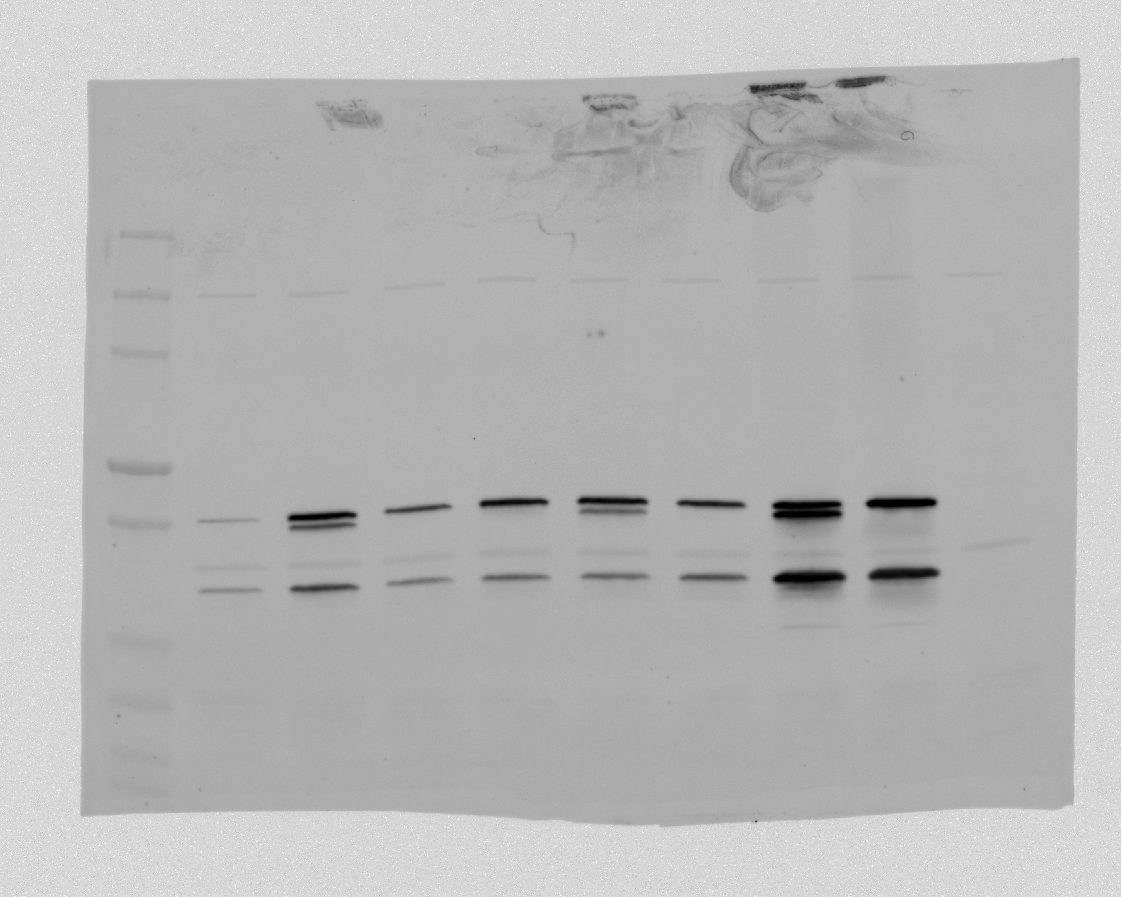

Supplement: Figure 2—source data 1. [file elife-70017-fig2-data1.zip › Figure 2 - Source Data 1/Variants (IRDye 800CW).tif]

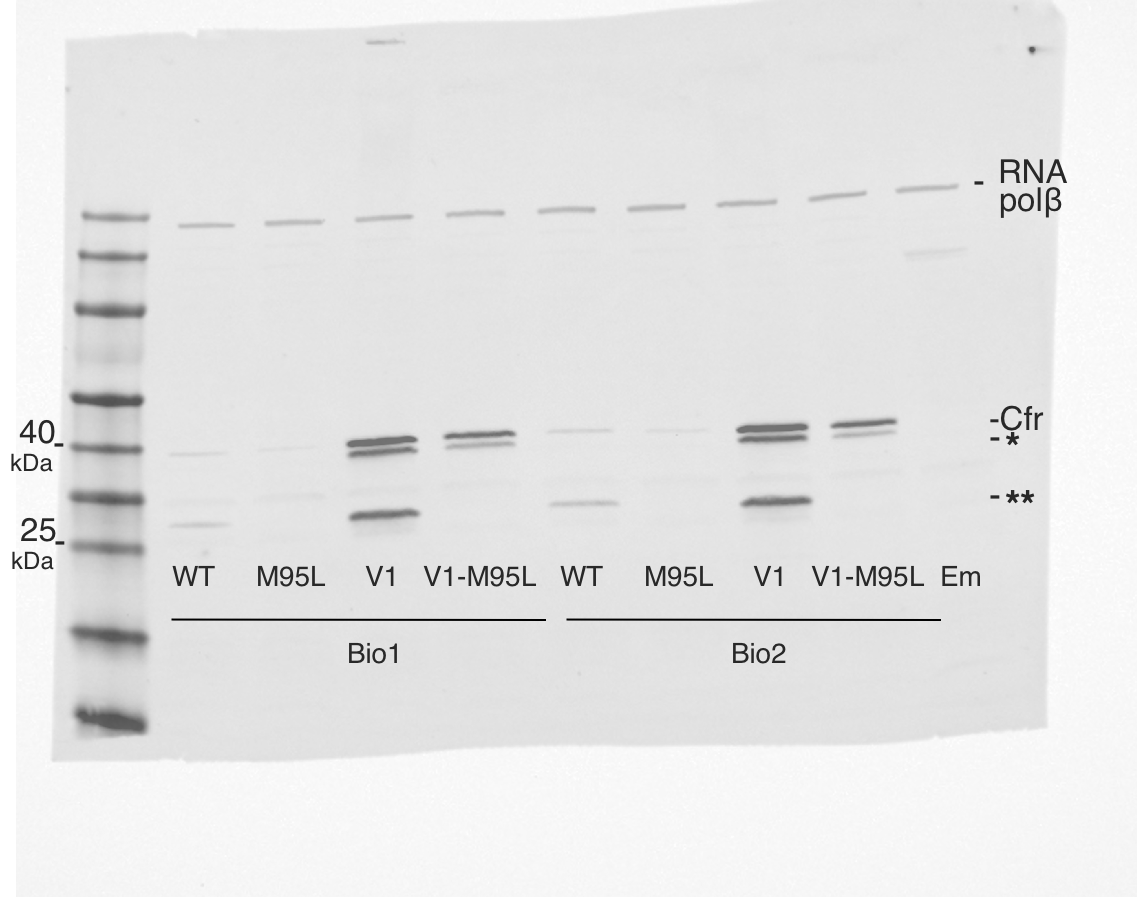

Supplement: Figure 2—source data 4. [file elife-70017-fig2-data4.zip › Figure 2 - Source Data 4 - figure supplement 3/M95L (Labels).tiff]

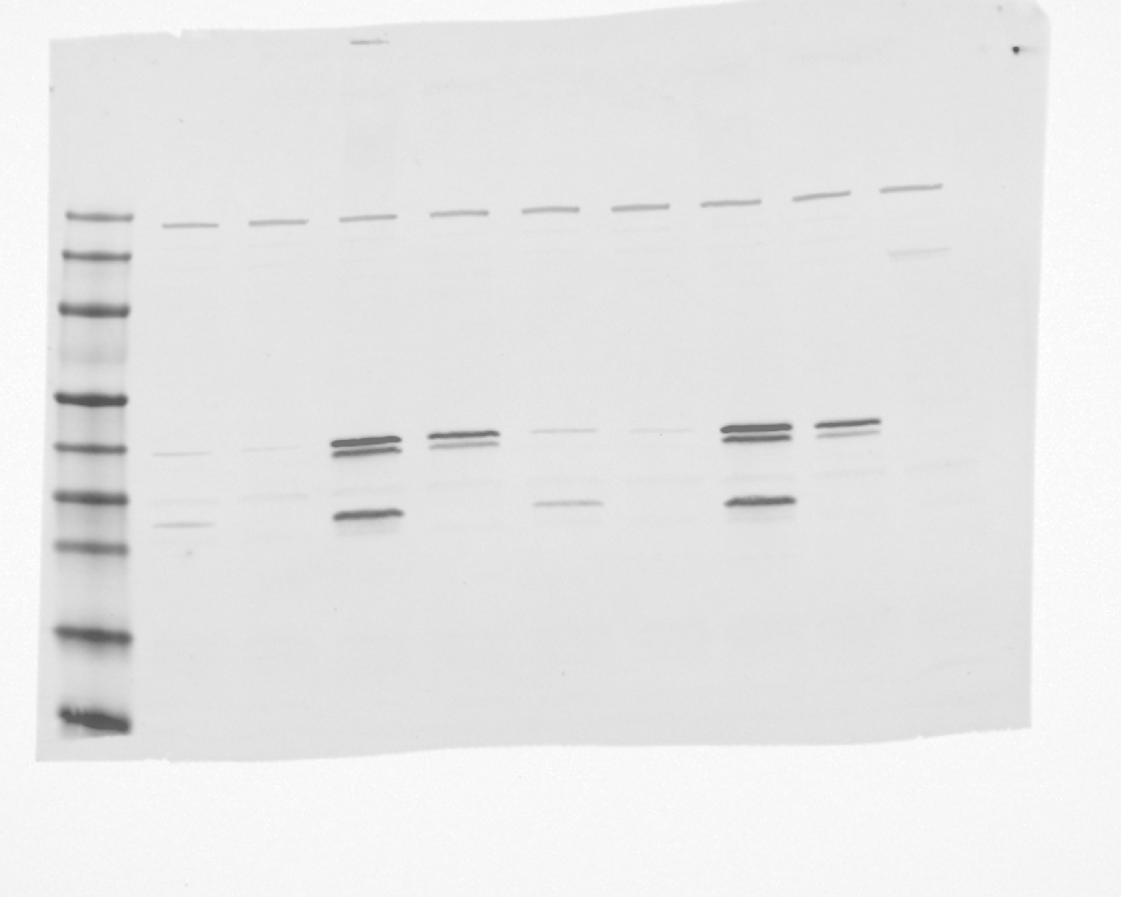

Supplement: Figure 2—source data 4. [file elife-70017-fig2-data4.zip › Figure 2 - Source Data 4 - figure supplement 3/M95L (Composite).tif]

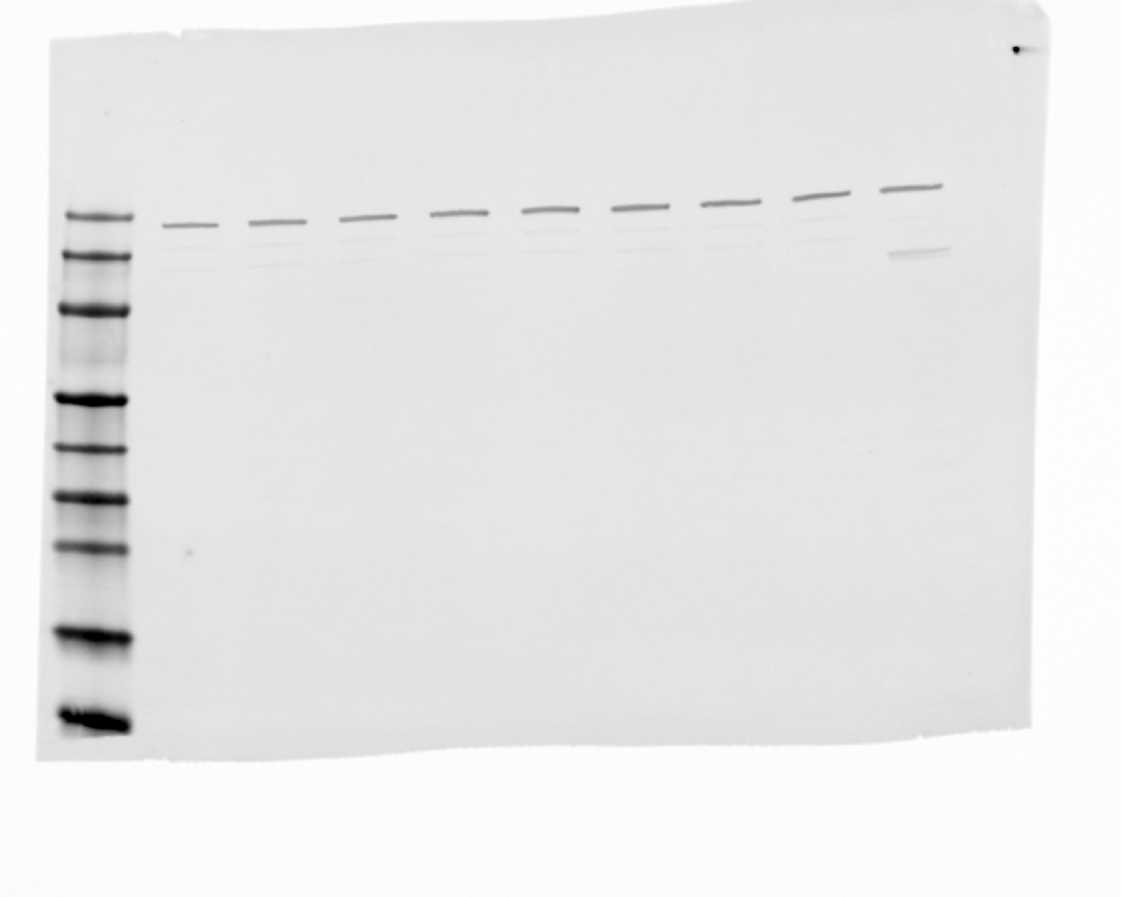

Supplement: Figure 2—source data 4. [file elife-70017-fig2-data4.zip › Figure 2 - Source Data 4 - figure supplement 3/M95L (DyLight 680).tif]

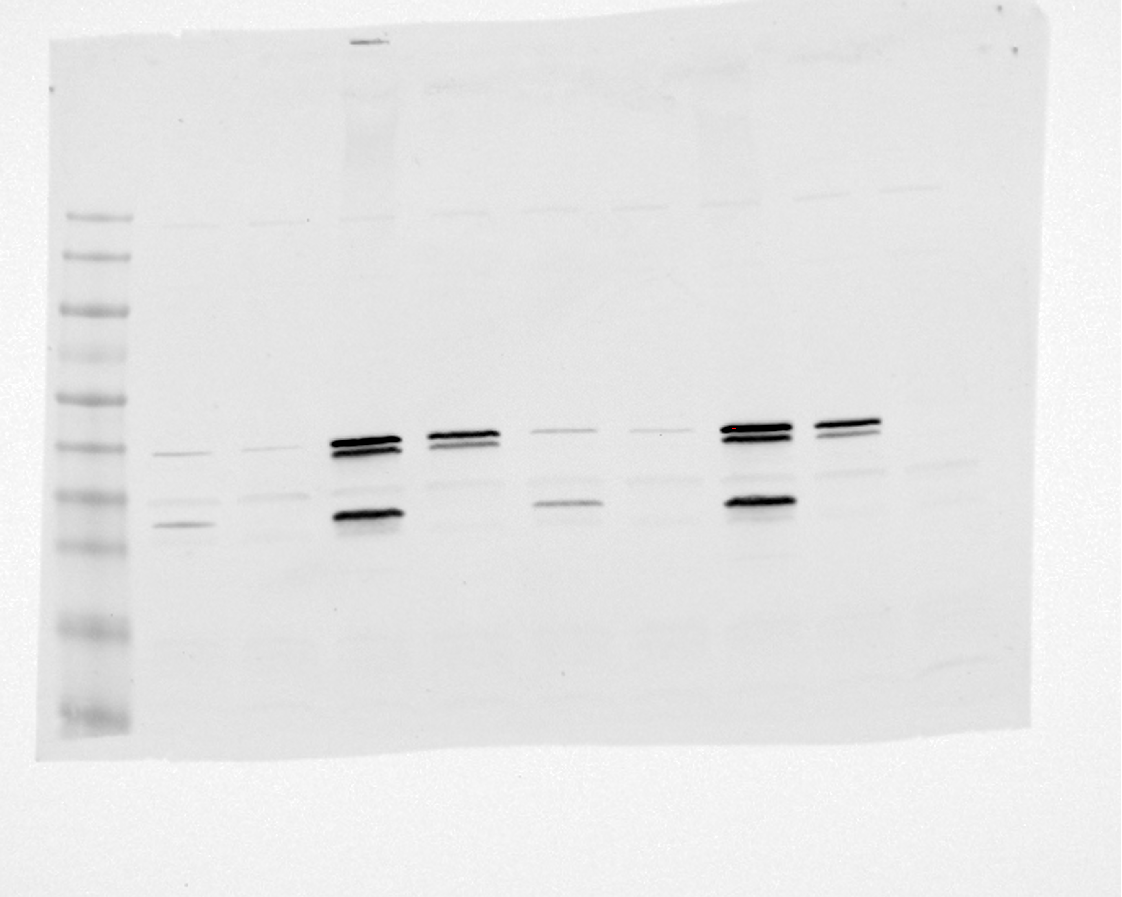

Supplement: Figure 2—source data 4. [file elife-70017-fig2-data4.zip › Figure 2 - Source Data 4 - figure supplement 3/M95L (IRDye 800CW).tif]

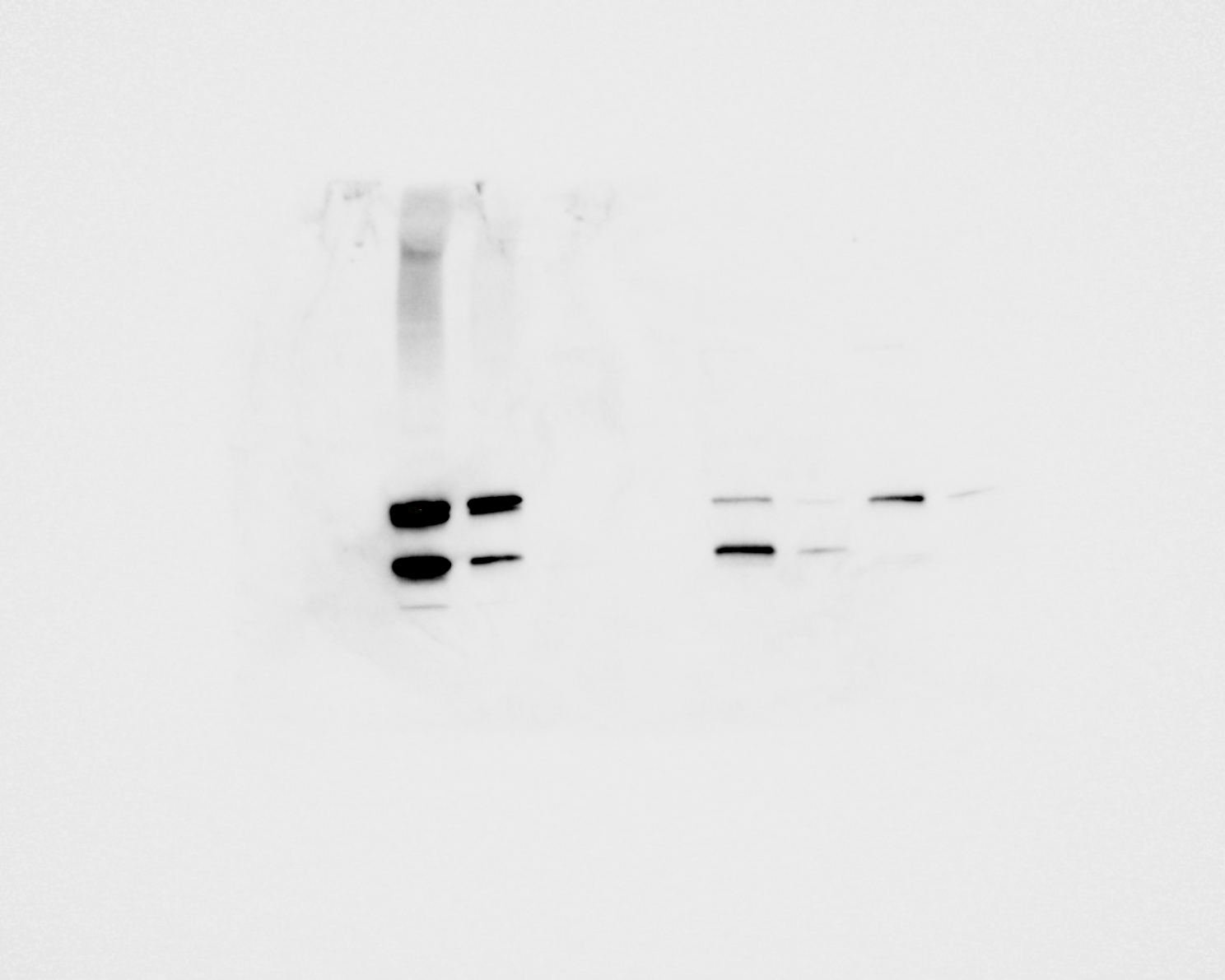

Supplement: Figure 2—source data 5. [file elife-70017-fig2-data5.zip › Figure 2 - Source Data 5 - figure supplement 3/Truncation Constructs (Chemiluminescence).tif]

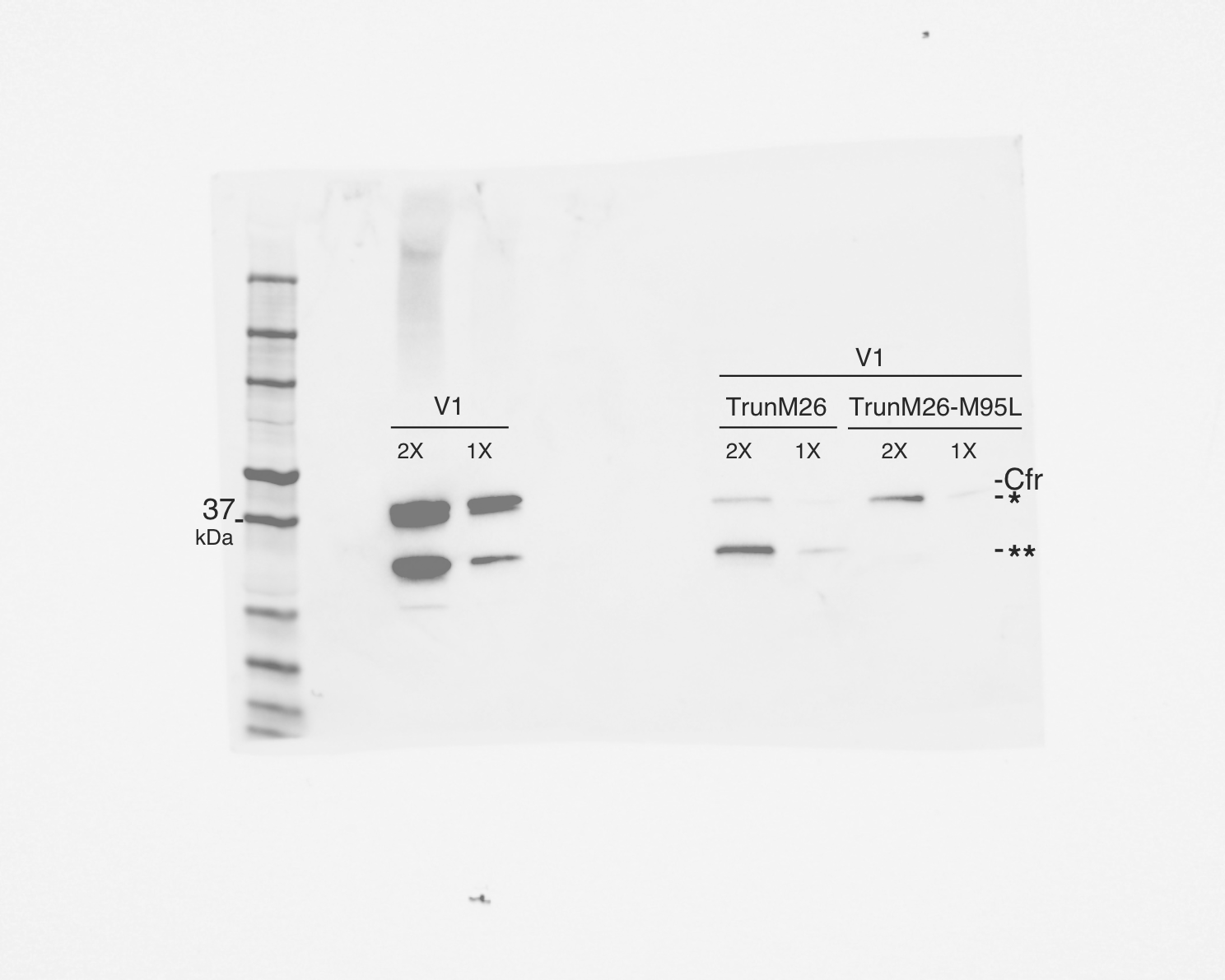

Supplement: Figure 2—source data 5. [file elife-70017-fig2-data5.zip › Figure 2 - Source Data 5 - figure supplement 3/Truncation Constructs (Labels).tiff]

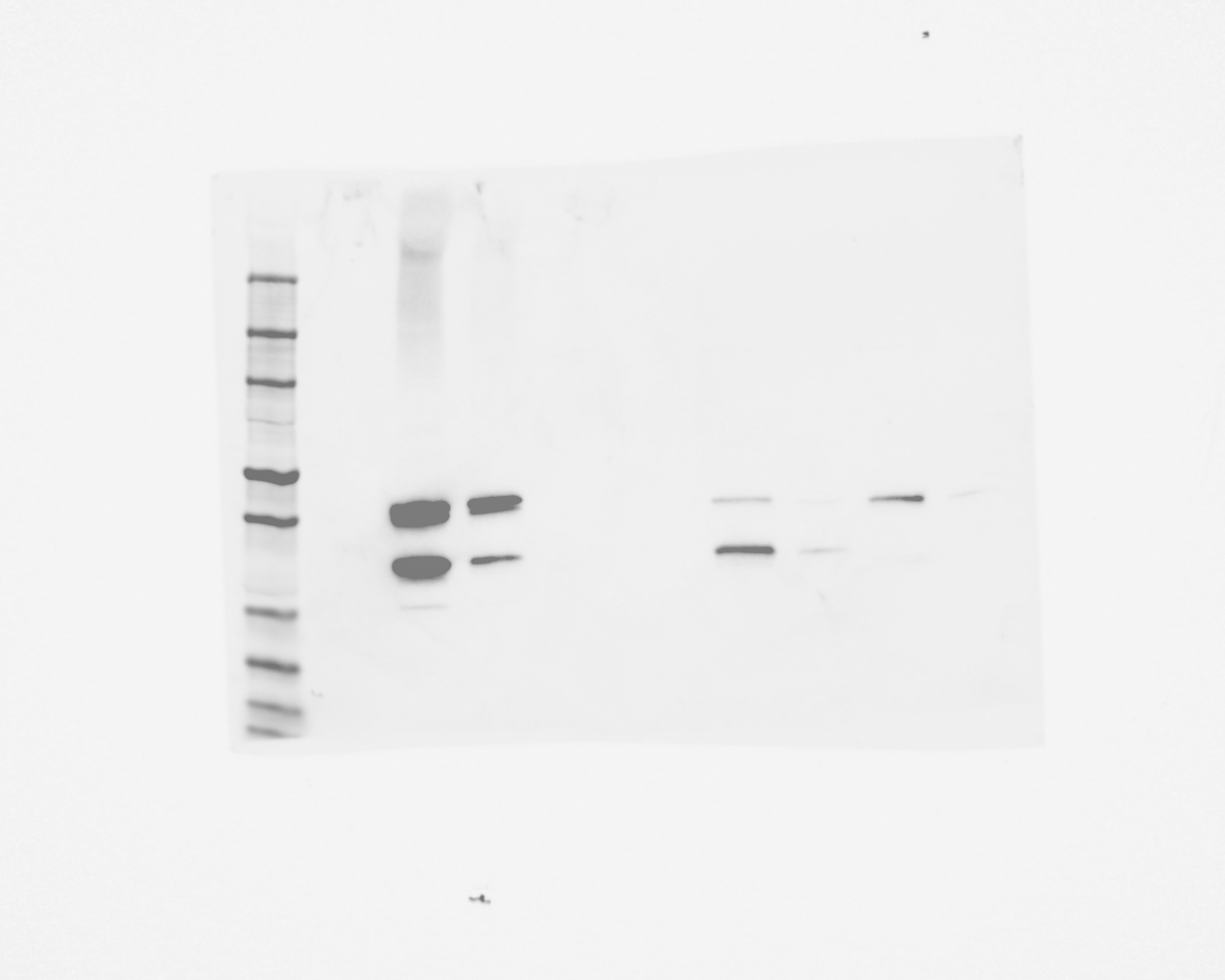

Supplement: Figure 2—source data 5. [file elife-70017-fig2-data5.zip › Figure 2 - Source Data 5 - figure supplement 3/Truncation Constructs (Composite).tif]

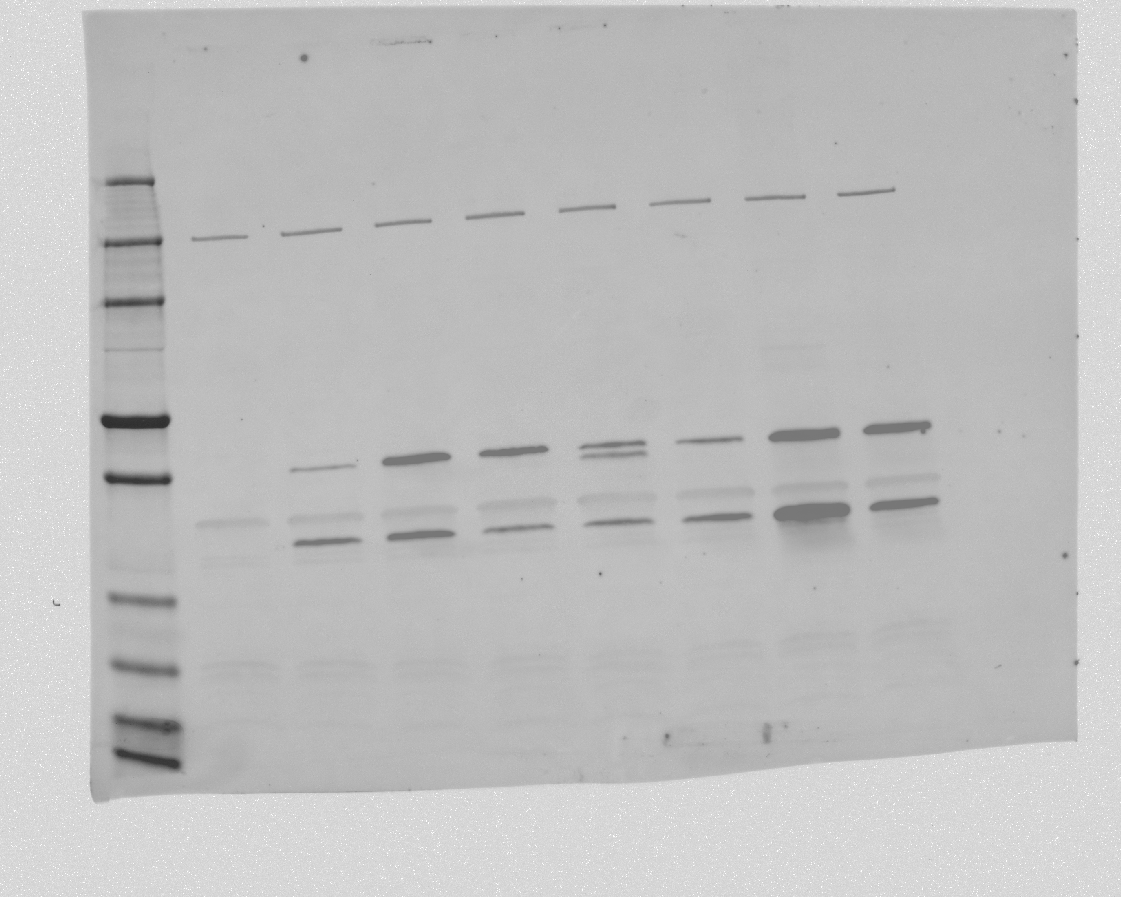

Supplement: Figure 3—source data 1. [file elife-70017-fig3-data1.zip › Figure 3 - Source Data 1/Mutants (Composite).tif]

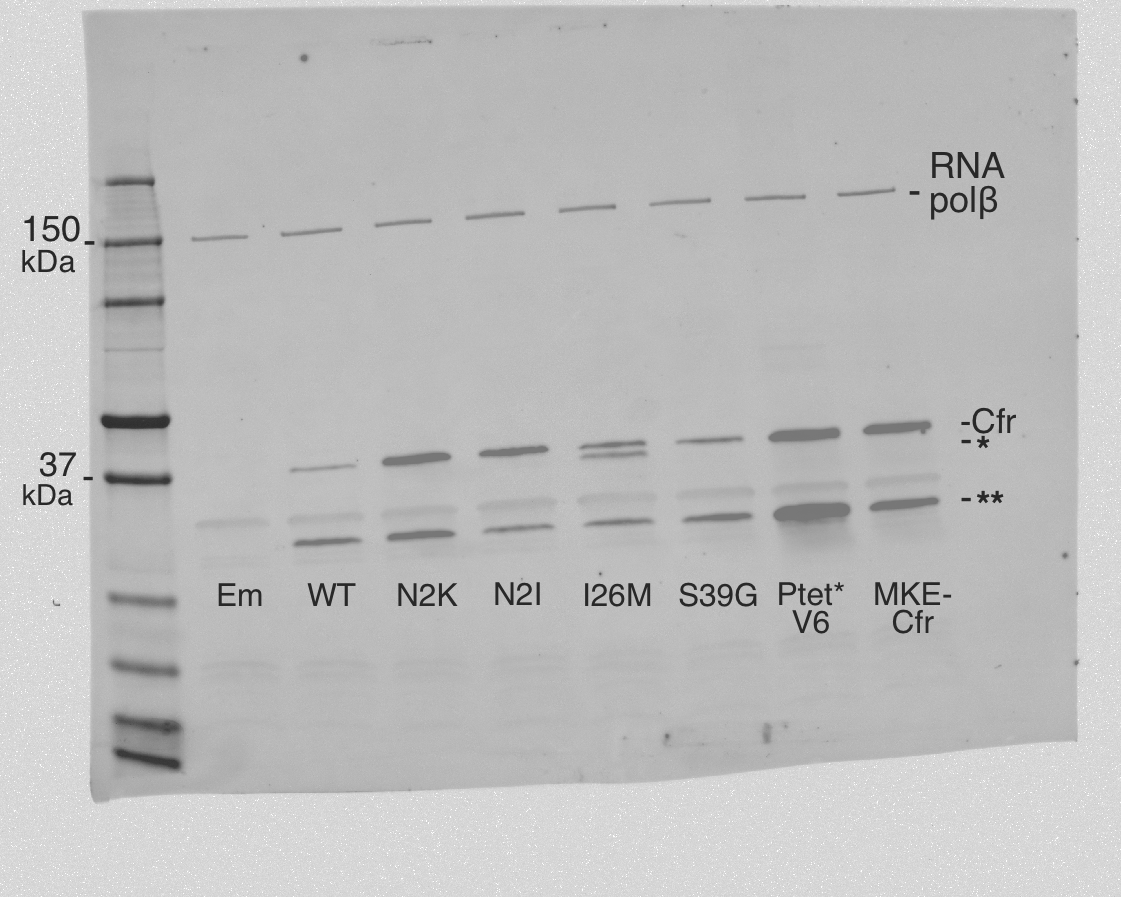

Supplement: Figure 3—source data 1. [file elife-70017-fig3-data1.zip › Figure 3 - Source Data 1/Mutants (Labels).tiff]

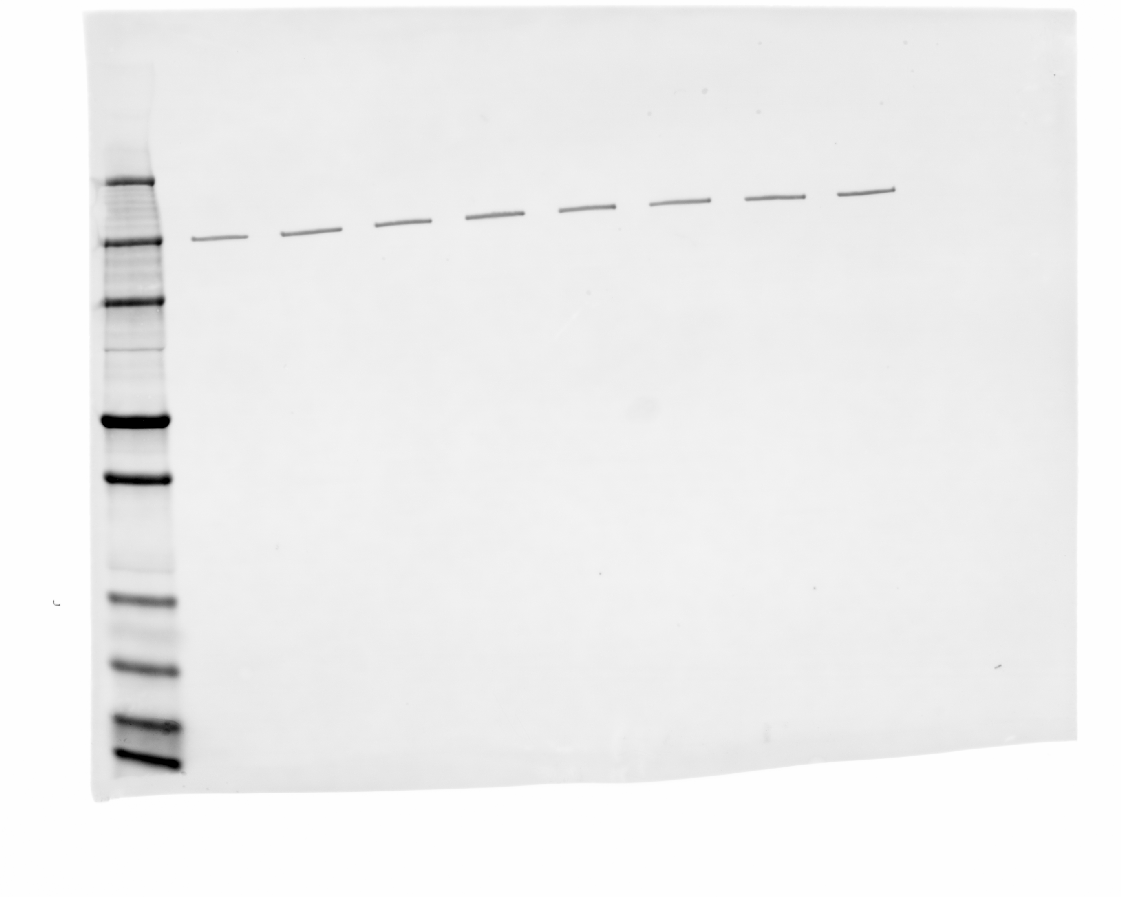

Supplement: Figure 3—source data 1. [file elife-70017-fig3-data1.zip › Figure 3 - Source Data 1/Mutants (DyLight 680).tif]

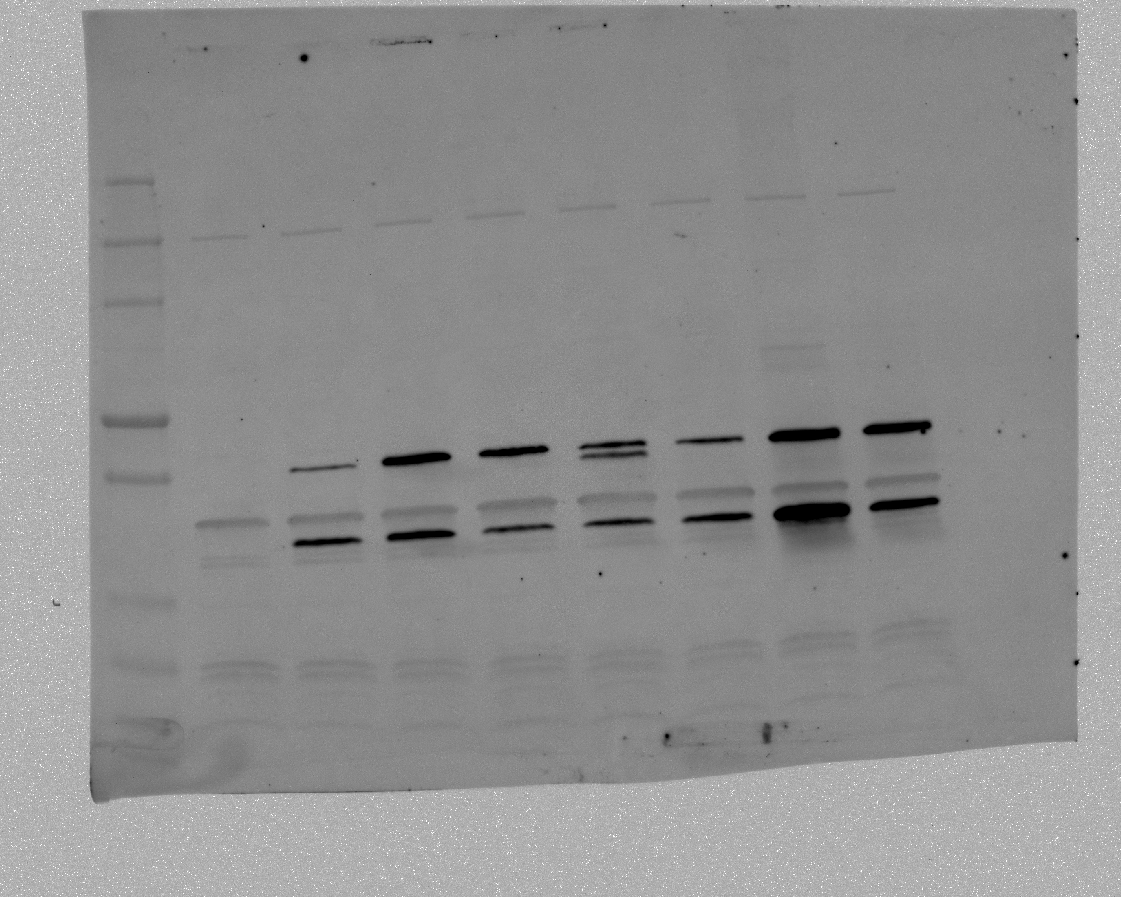

Supplement: Figure 3—source data 1. [file elife-70017-fig3-data1.zip › Figure 3 - Source Data 1/Mutants (IRDye 800CW).tif]

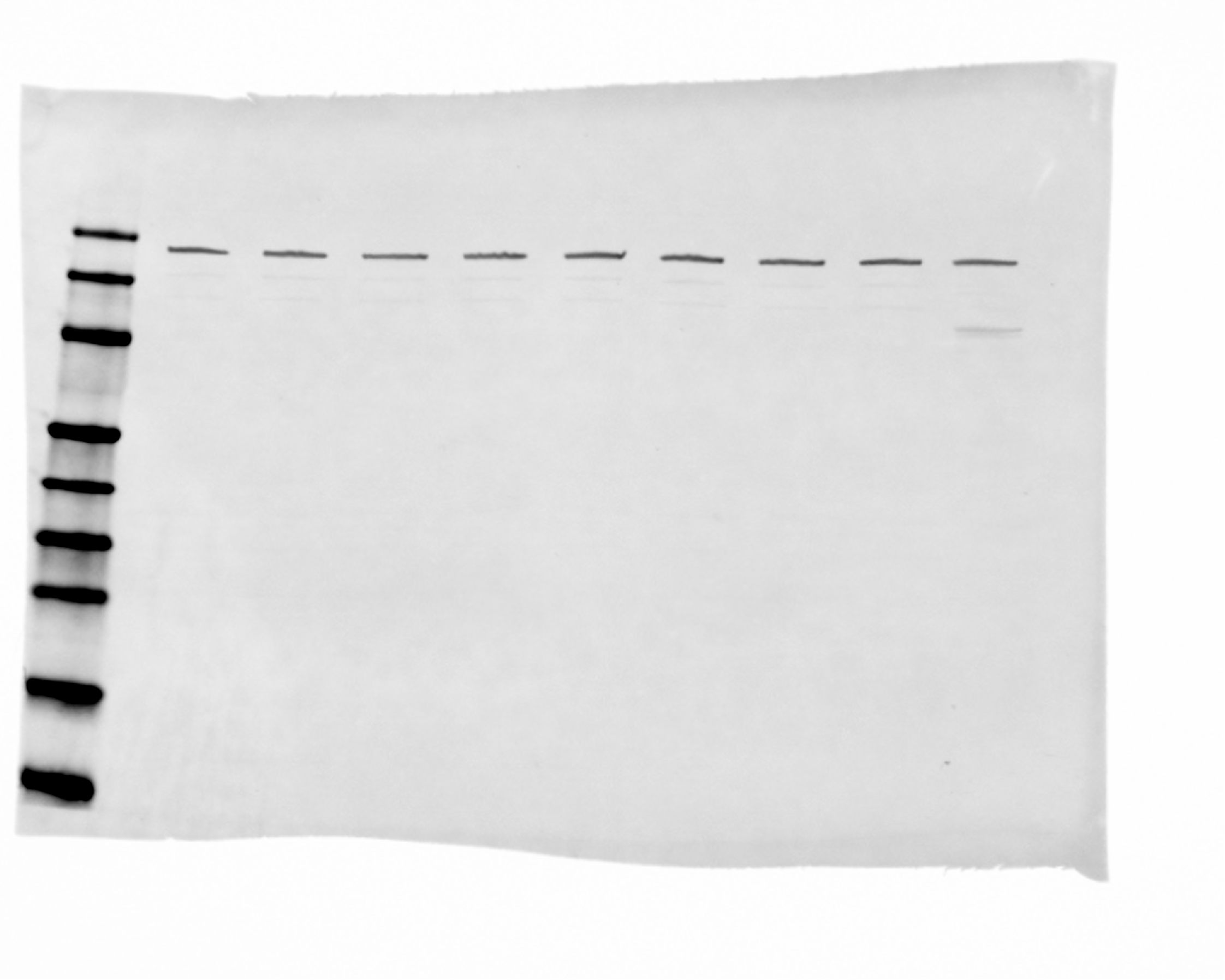

Supplement: Figure 3—source data 4. [file elife-70017-fig3-data4.zip › Figure 3 - Source Data 4 - figure supplement 2/C338A (DyLight 680).tif]

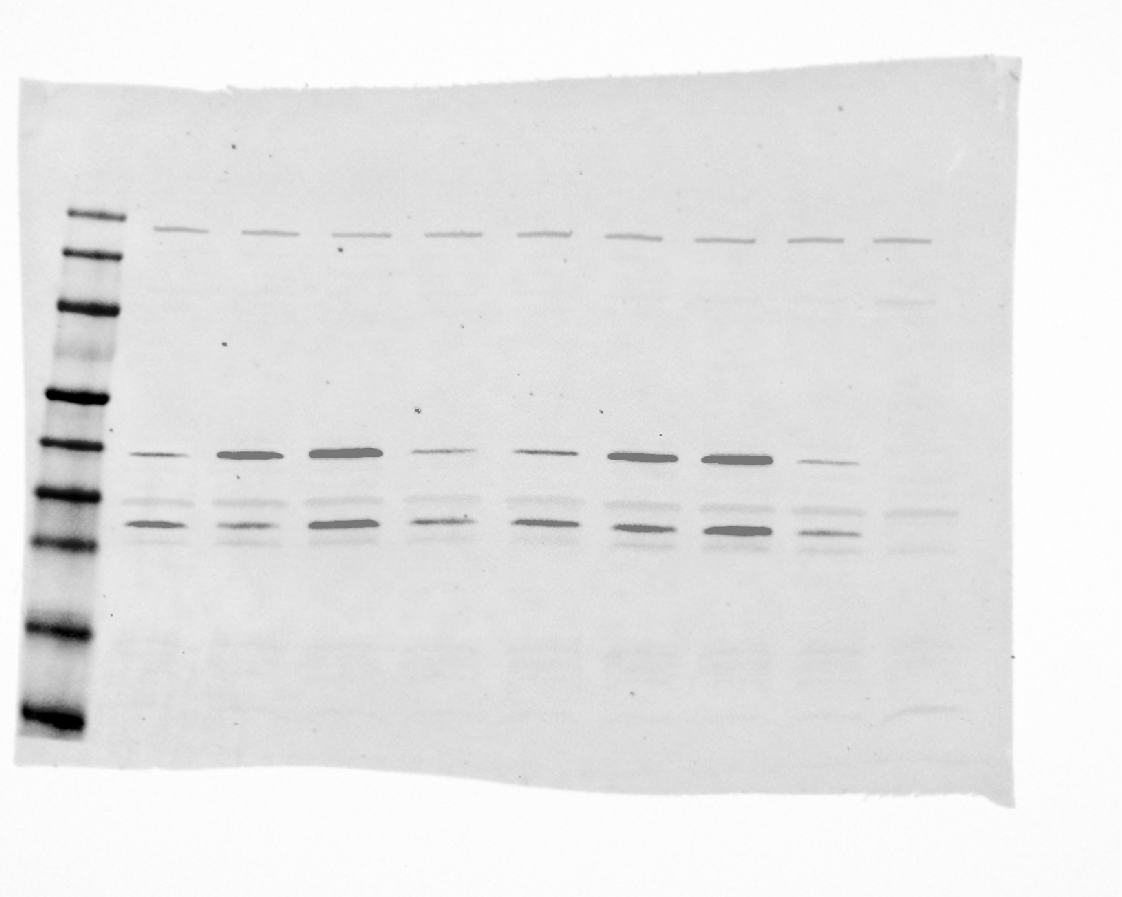

Supplement: Figure 3—source data 4. [file elife-70017-fig3-data4.zip › Figure 3 - Source Data 4 - figure supplement 2/C338A (Composite).tif]

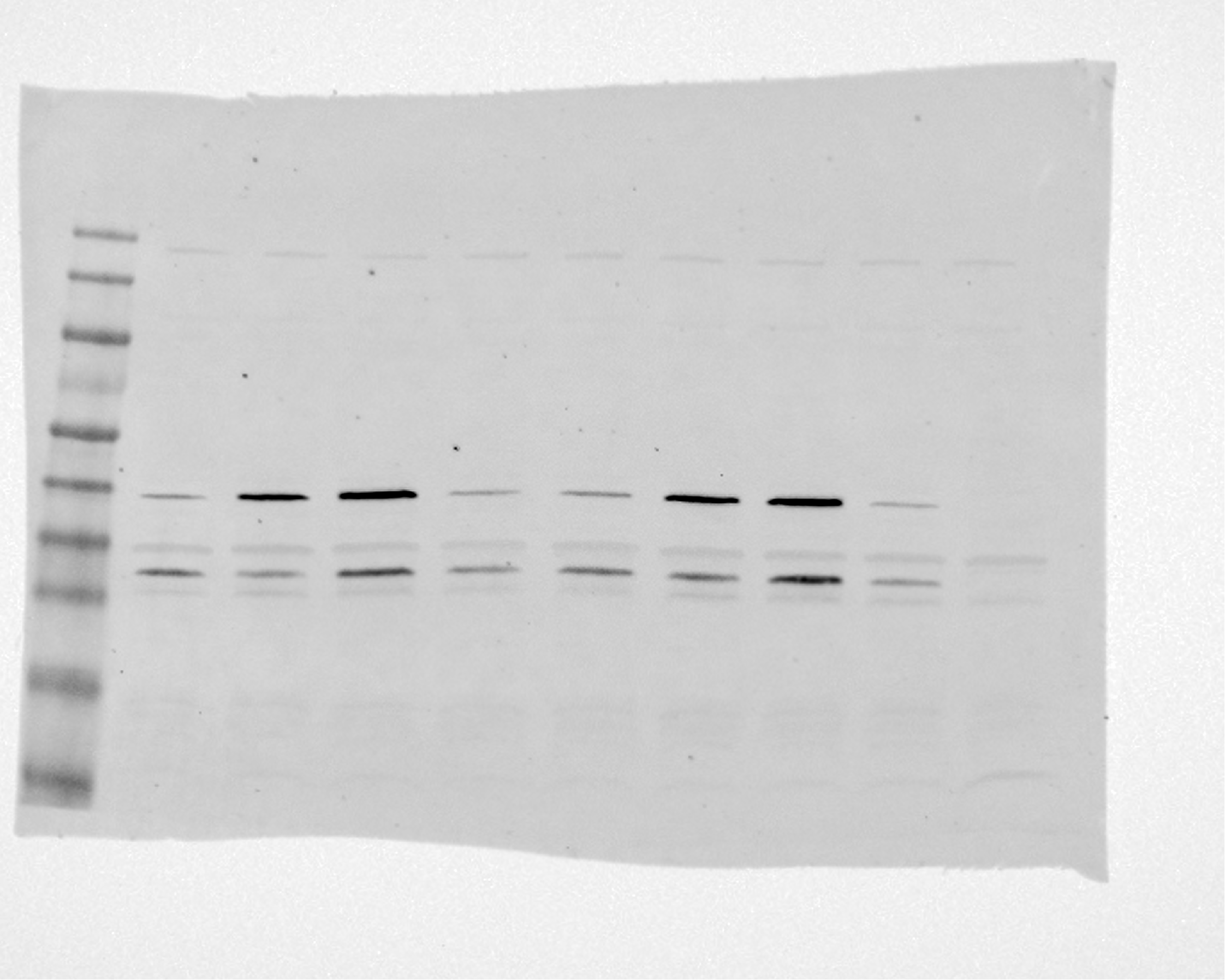

Supplement: Figure 3—source data 4. [file elife-70017-fig3-data4.zip › Figure 3 - Source Data 4 - figure supplement 2/C338A (IRDye 800CW).tif]

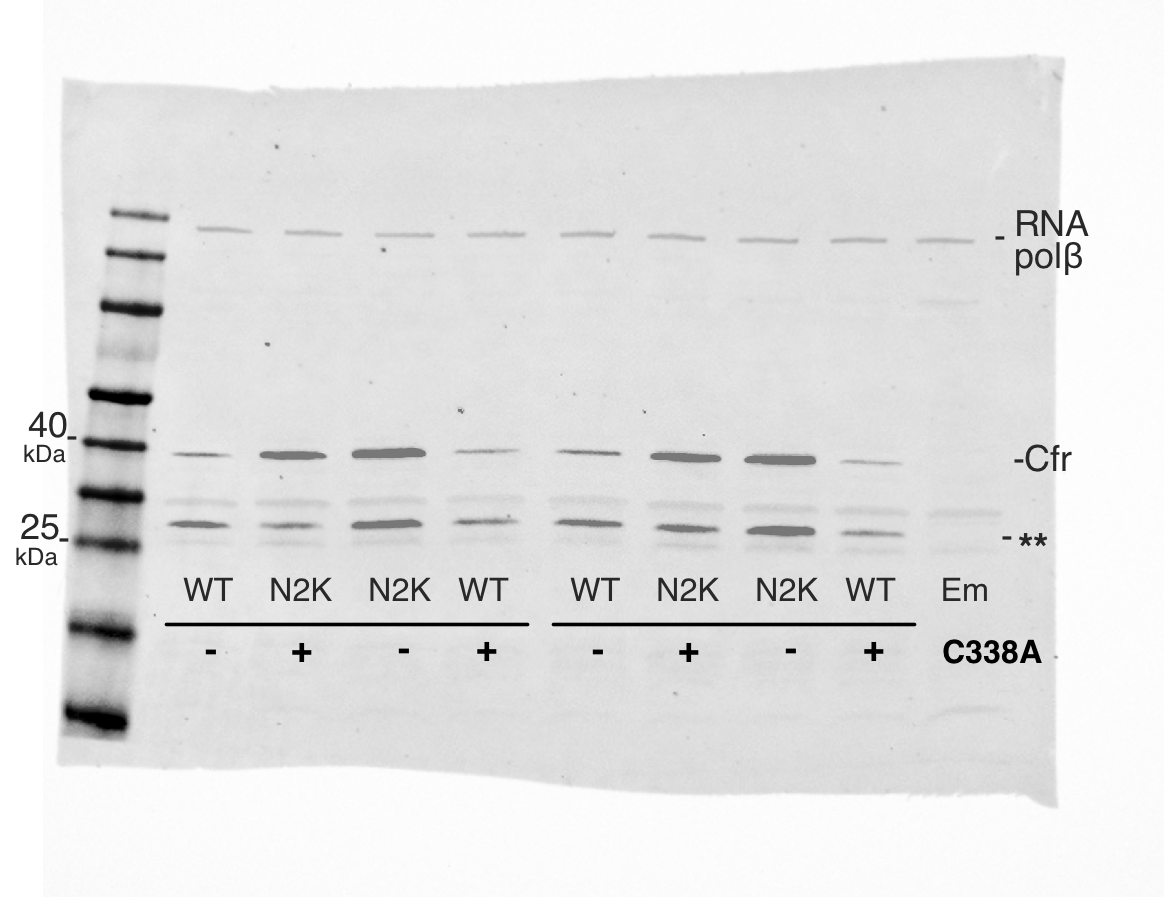

Supplement: Figure 3—source data 4. [file elife-70017-fig3-data4.zip › Figure 3 - Source Data 4 - figure supplement 2/C338A (Labels).tiff]

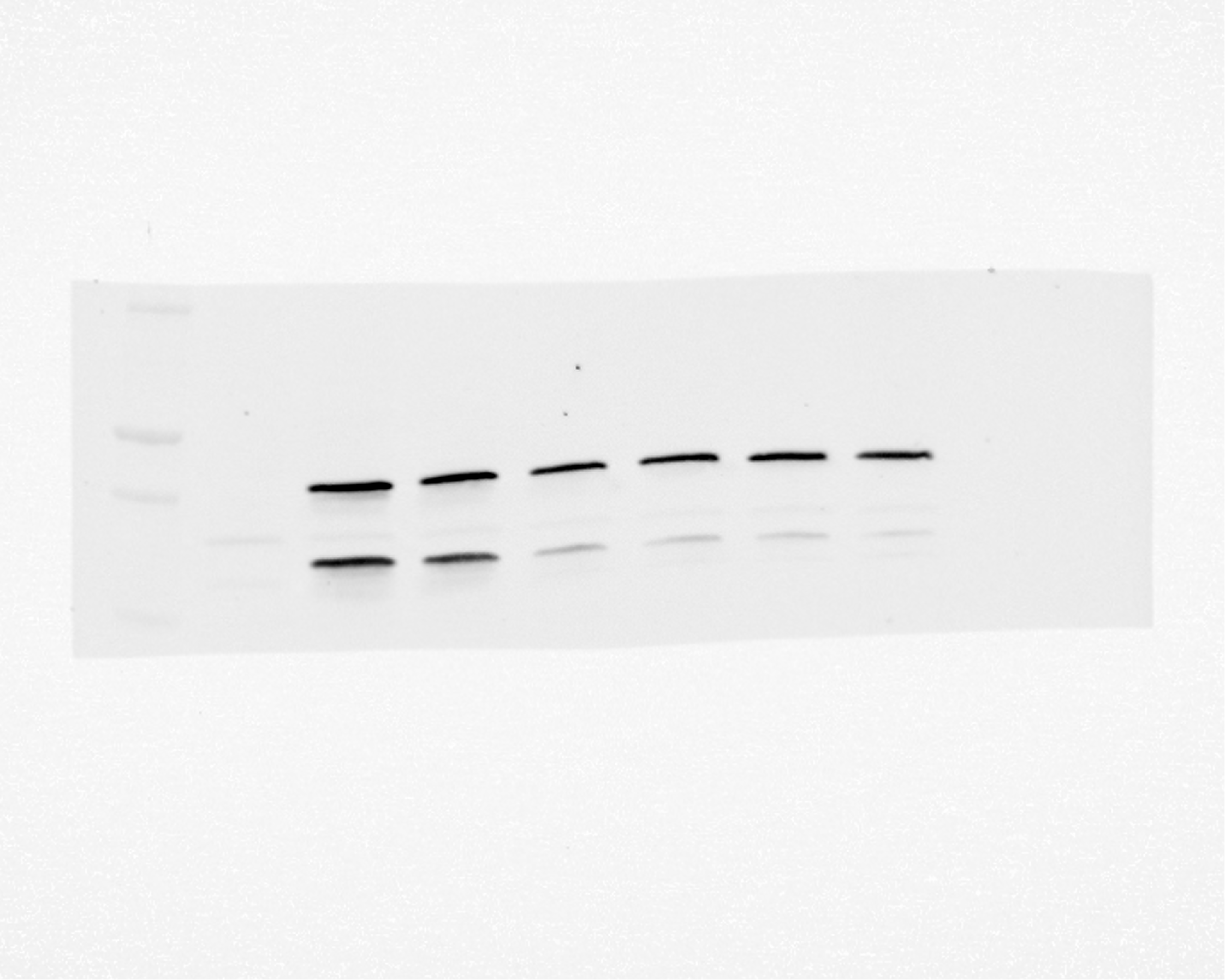

Supplement: Figure 4—source data 1. [file elife-70017-fig4-data1.zip › Figure 4 - Source Data 1/V3 (IRDye 800CW).tif]

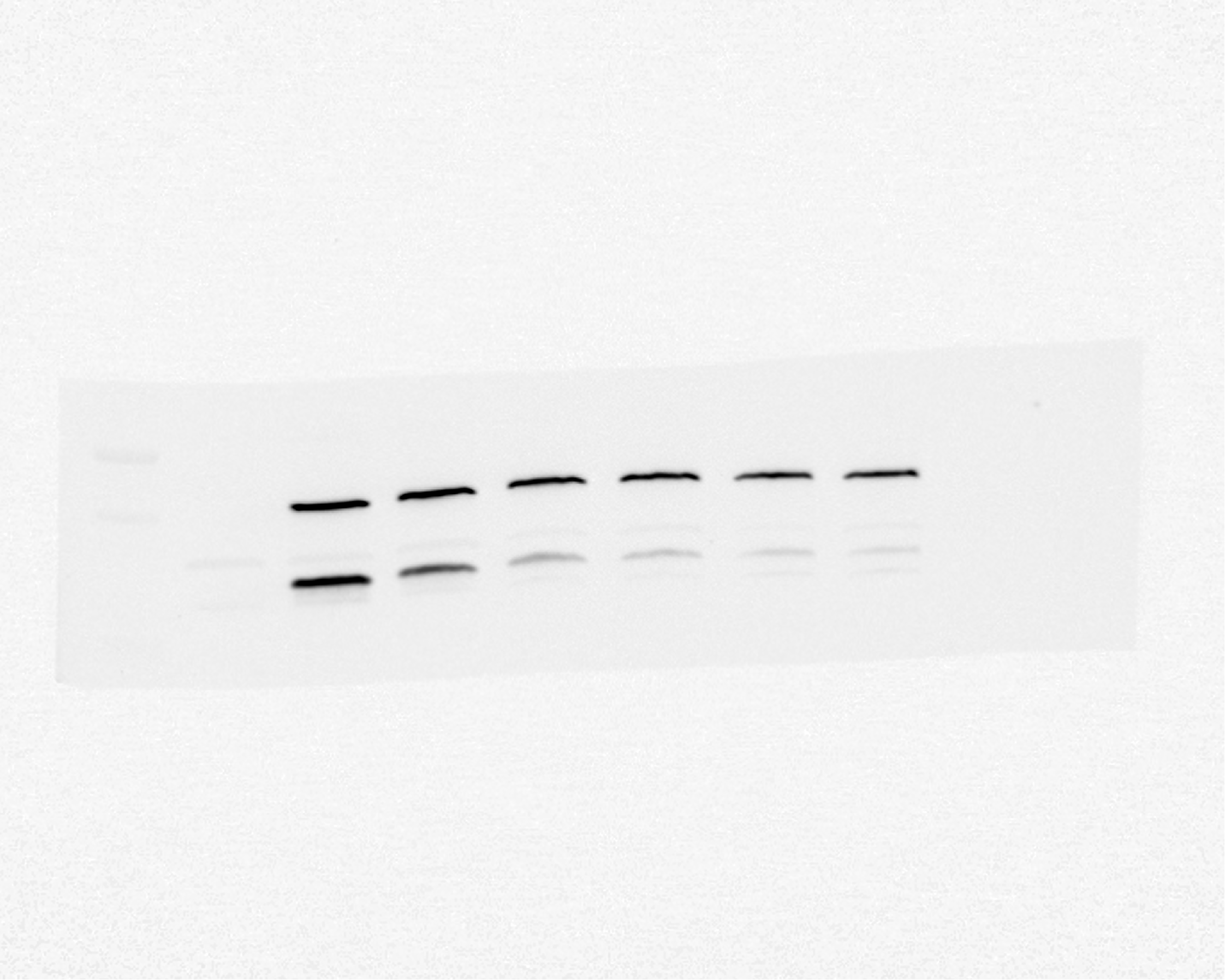

Supplement: Figure 4—source data 1. [file elife-70017-fig4-data1.zip › Figure 4 - Source Data 1/N2K (IRDye 800CW).tif]

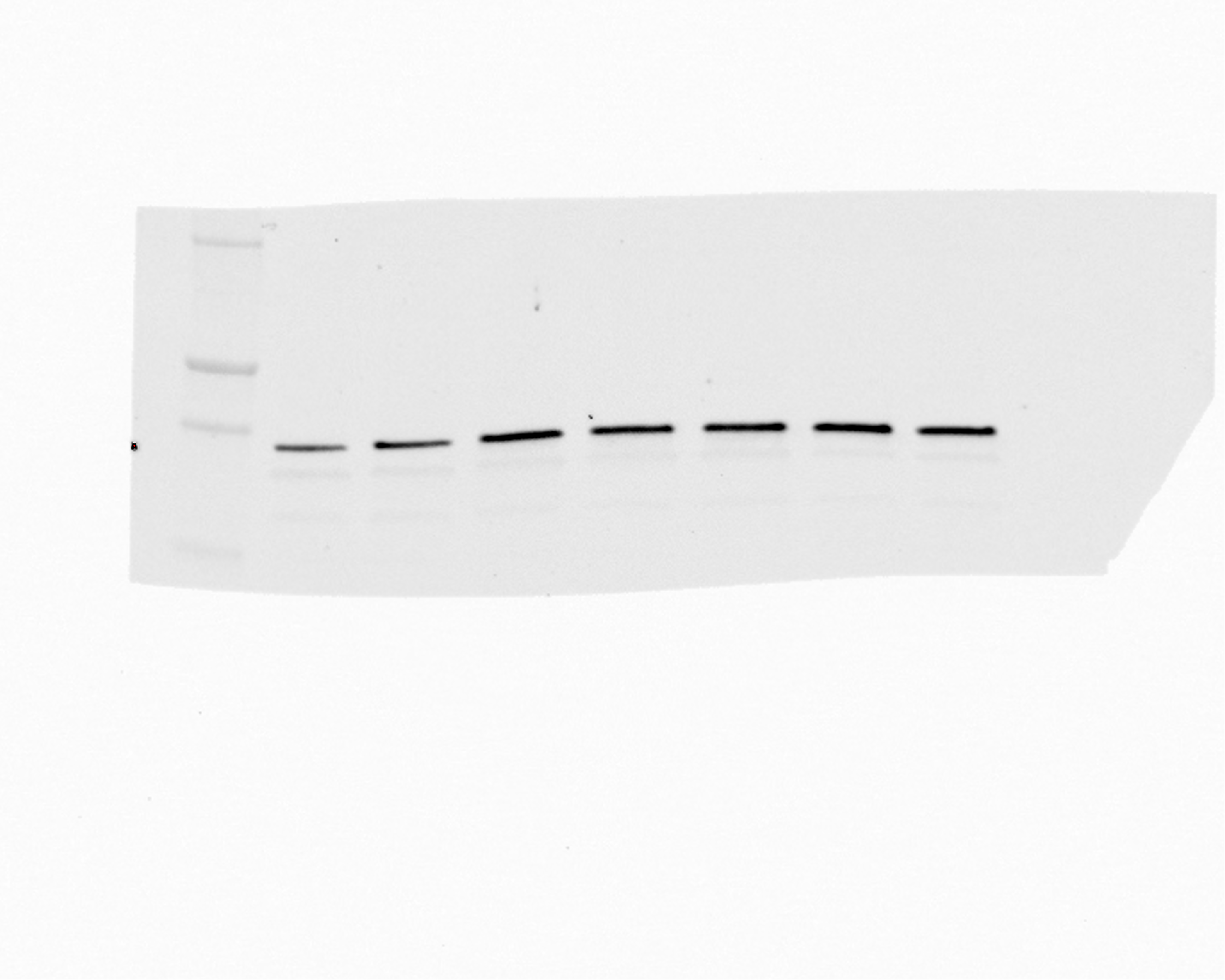

Supplement: Figure 4—source data 1. [file elife-70017-fig4-data1.zip › Figure 4 - Source Data 1/GAPDH (IRDye 800CW).tif]

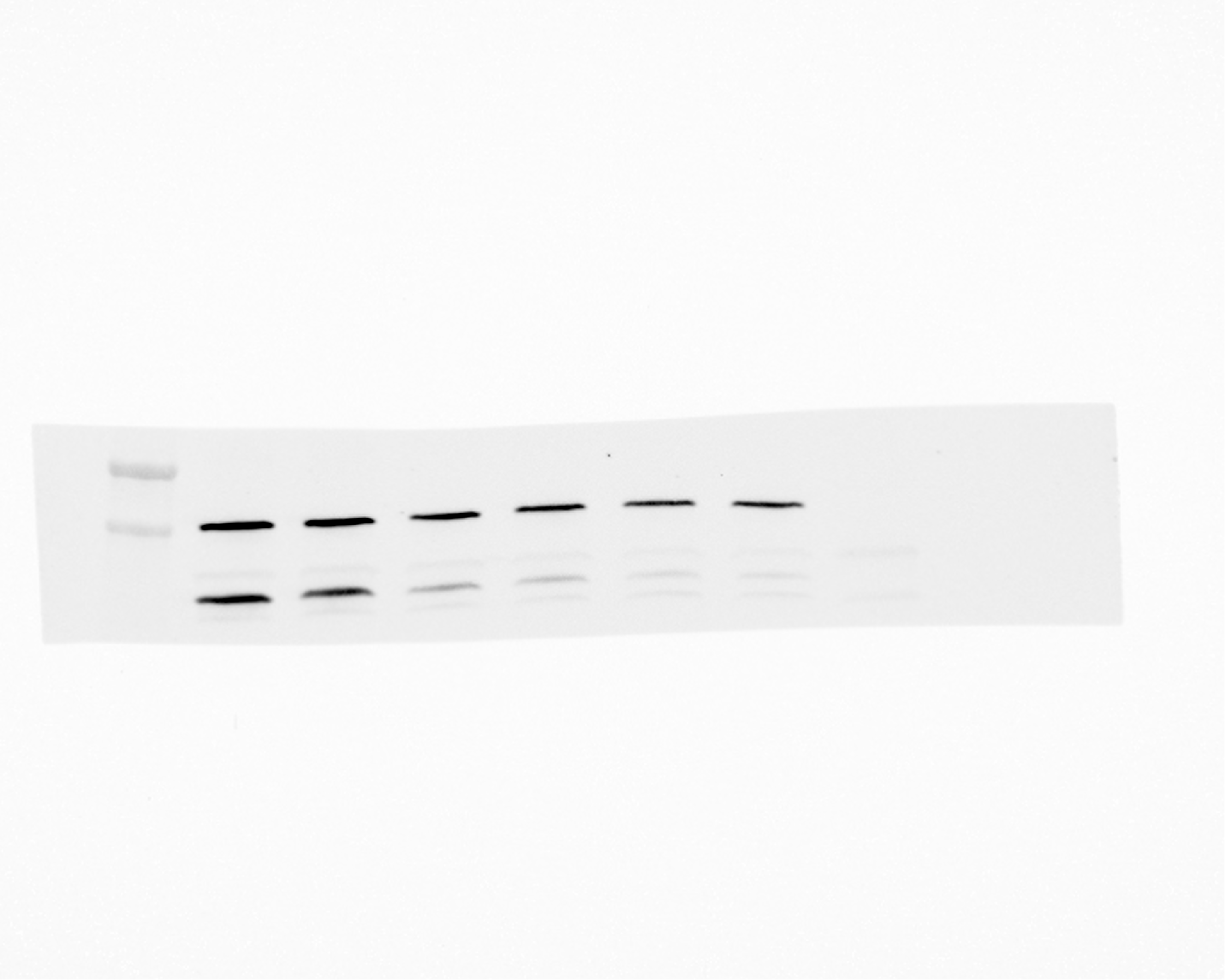

Supplement: Figure 4—source data 1. [file elife-70017-fig4-data1.zip › Figure 4 - Source Data 1/N2I (IRDye 800CW).tif]

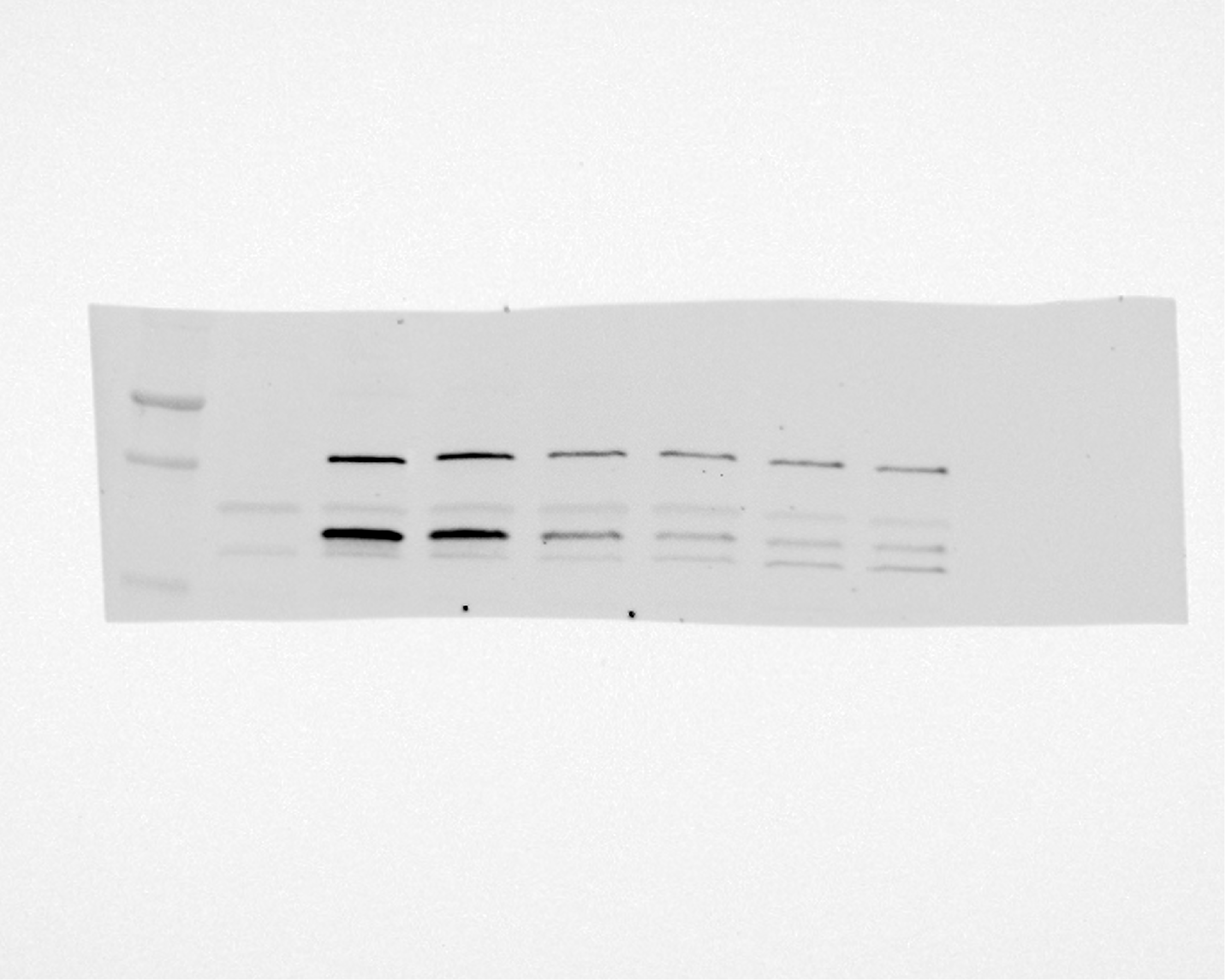

Supplement: Figure 4—source data 1. [file elife-70017-fig4-data1.zip › Figure 4 - Source Data 1/WT (IRDye 800CW).tif]

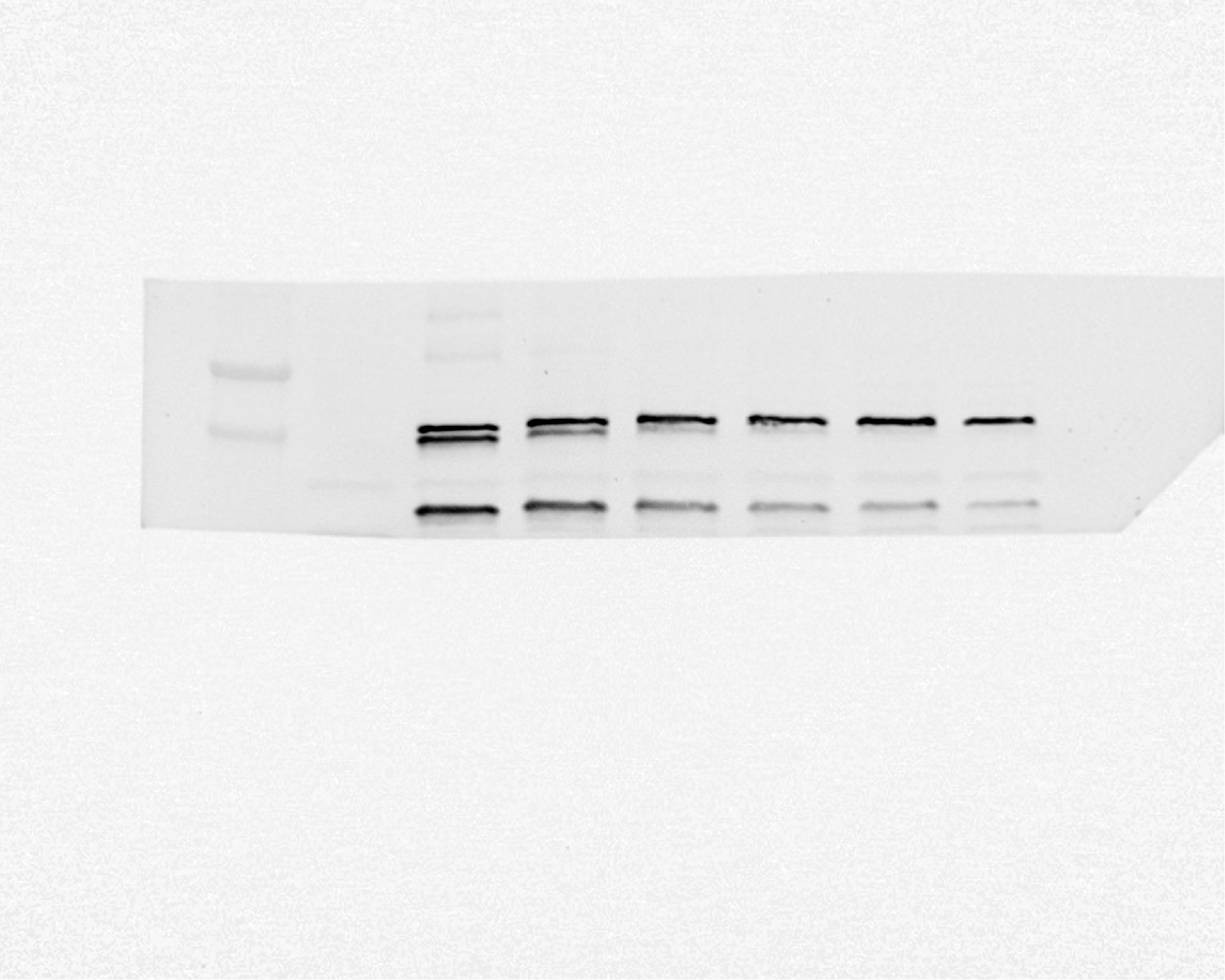

Supplement: Figure 4—source data 1. [file elife-70017-fig4-data1.zip › Figure 4 - Source Data 1/I26M (IRDye 800CW).tif]

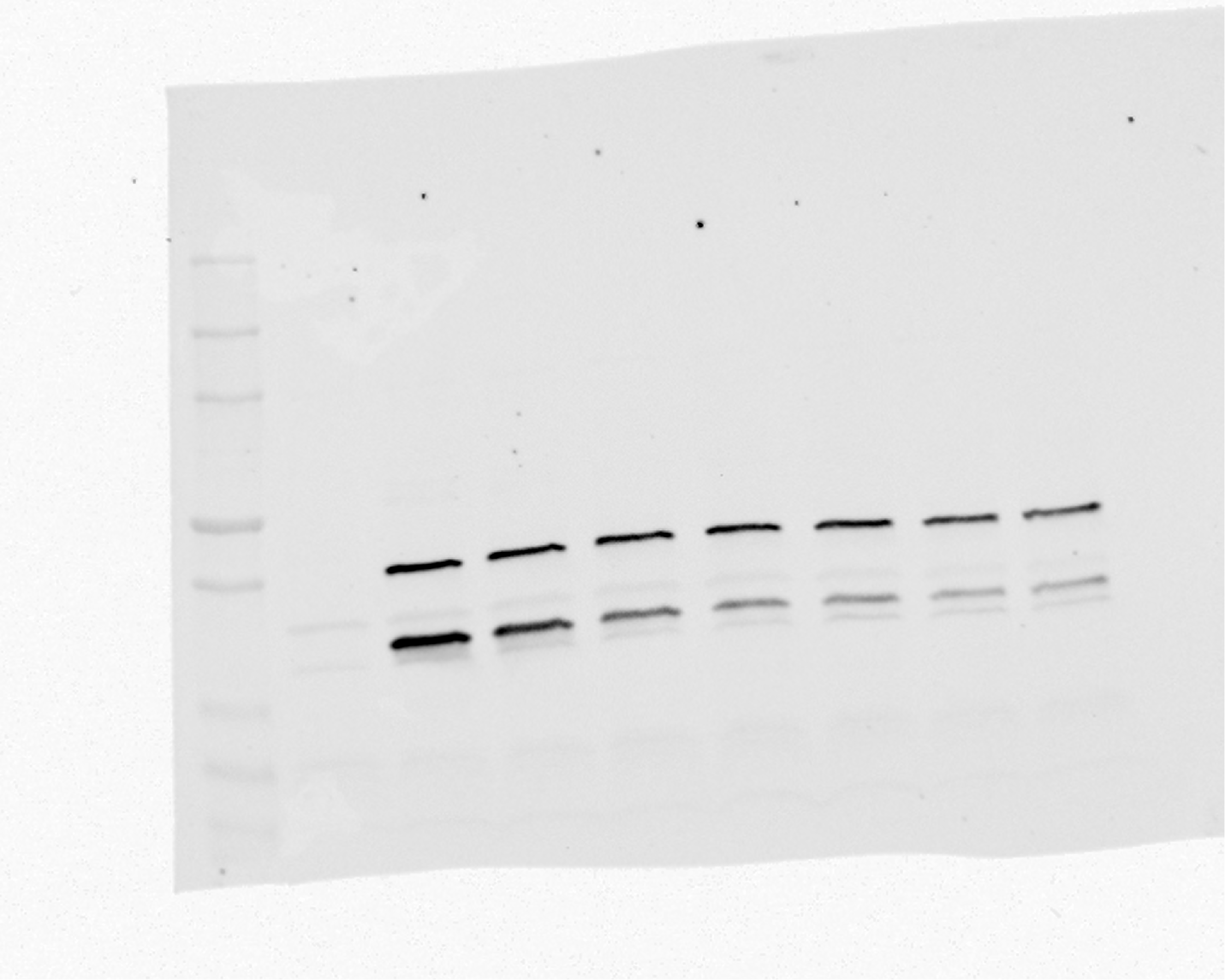

Supplement: Figure 4—source data 1. [file elife-70017-fig4-data1.zip › Figure 4 - Source Data 1/S39G (IRDye 800CW).tif]

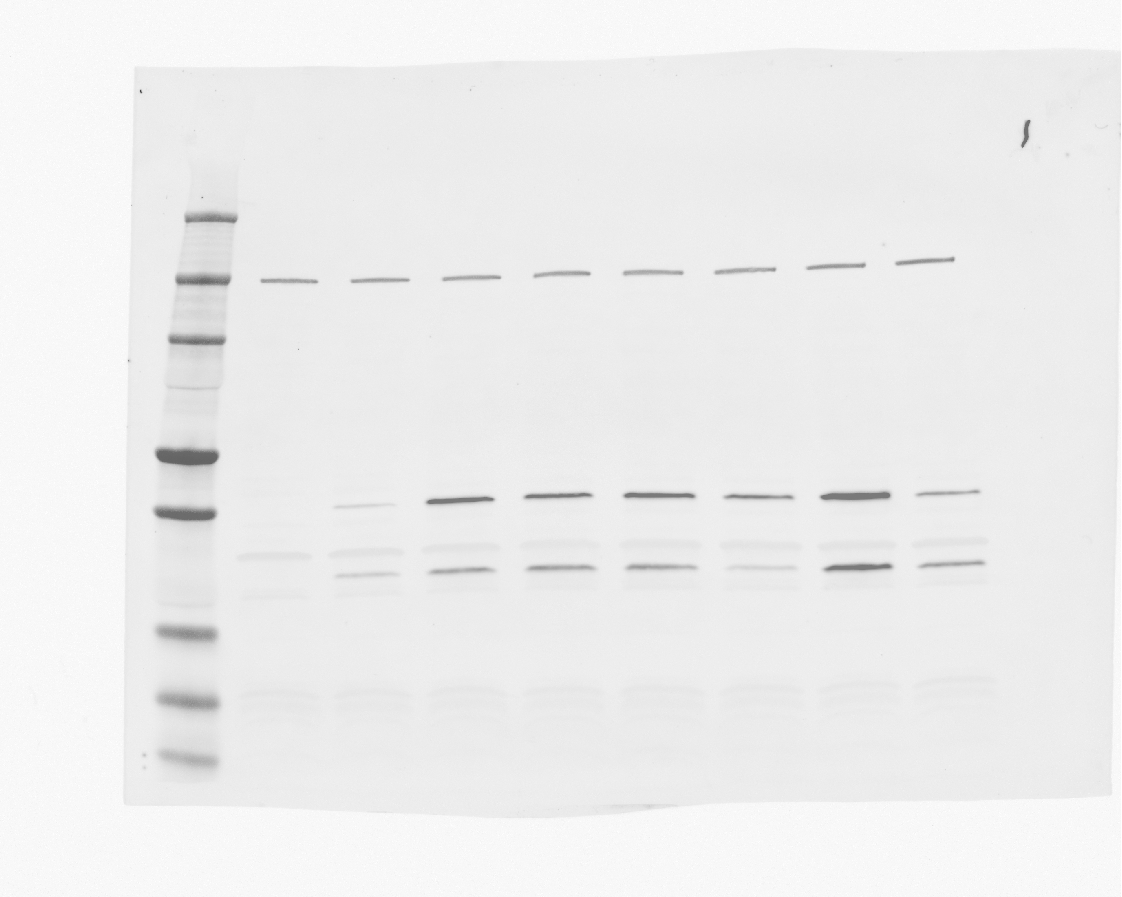

Supplement: Figure 4—source data 4. [file elife-70017-fig4-data4.zip › Figure 4 - Source Data 4 - figure supplement 2 /2nd Codon (Composite).tif]

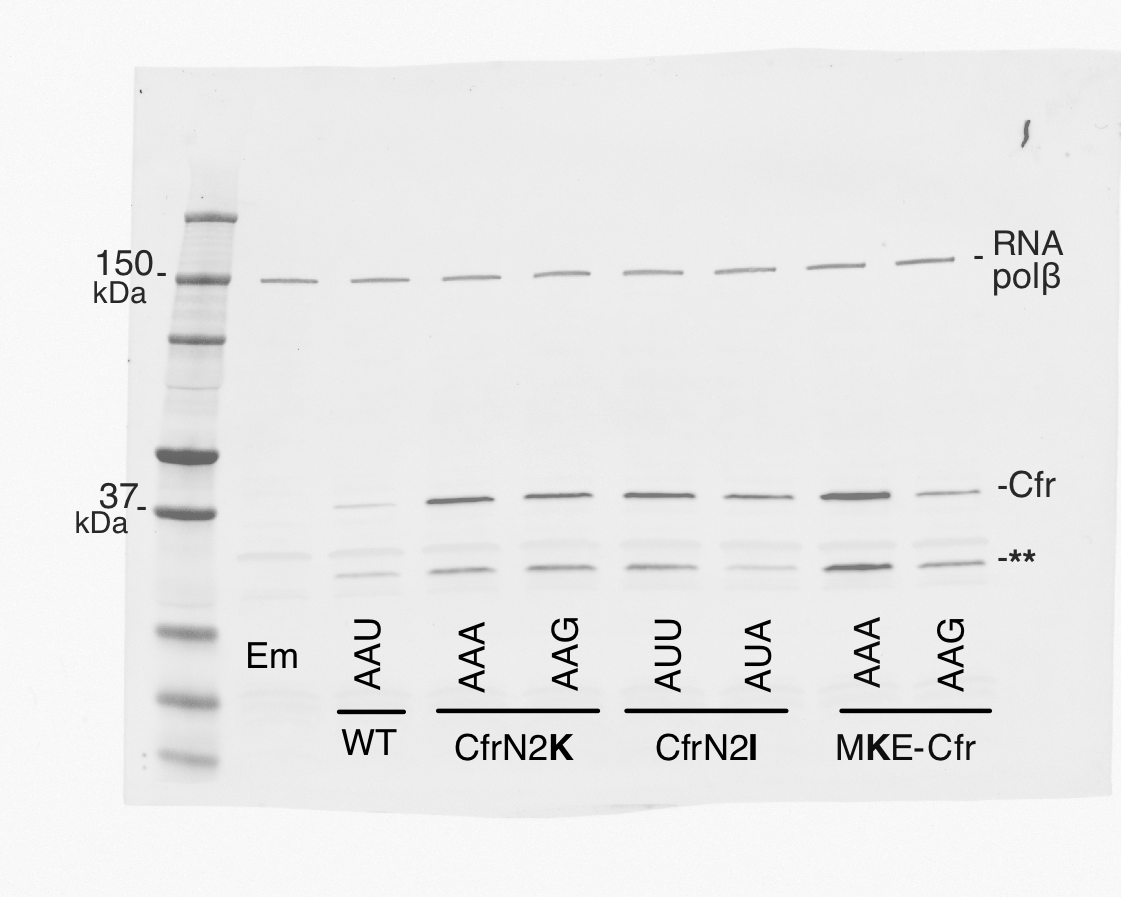

Supplement: Figure 4—source data 4. [file elife-70017-fig4-data4.zip › Figure 4 - Source Data 4 - figure supplement 2 /2nd Codon (Labels).tiff]

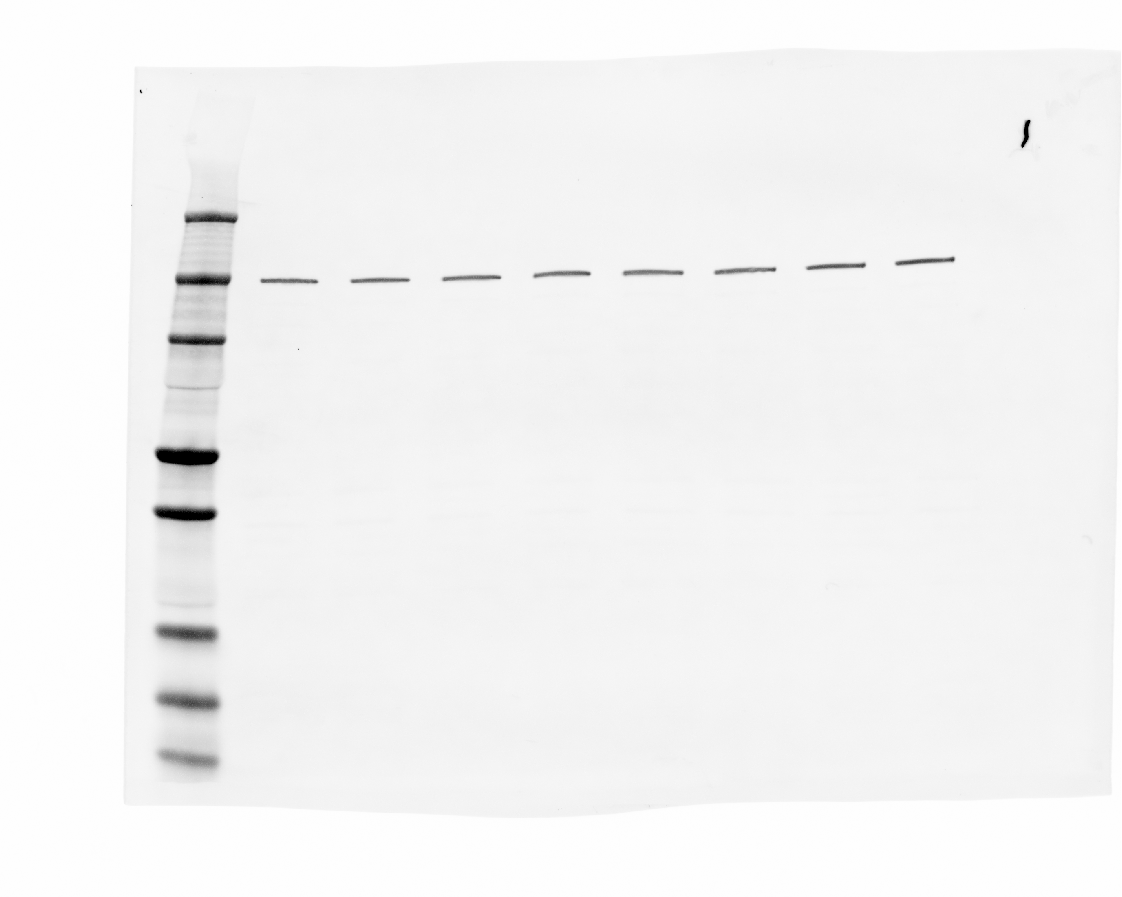

Supplement: Figure 4—source data 4. [file elife-70017-fig4-data4.zip › Figure 4 - Source Data 4 - figure supplement 2 /2nd Codon (DyLight 680).tif]

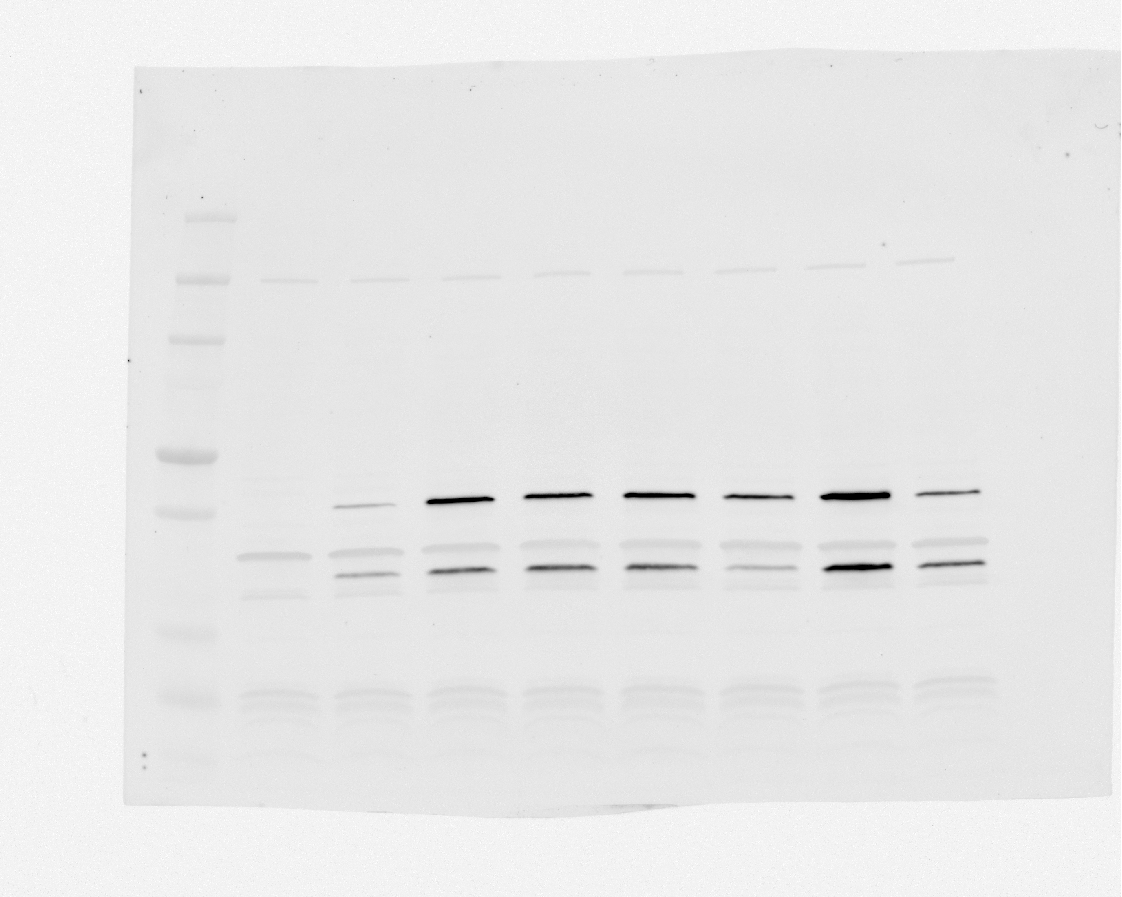

Supplement: Figure 4—source data 4. [file elife-70017-fig4-data4.zip › Figure 4 - Source Data 4 - figure supplement 2 /2nd Codon (IRDye 800CW).tif]

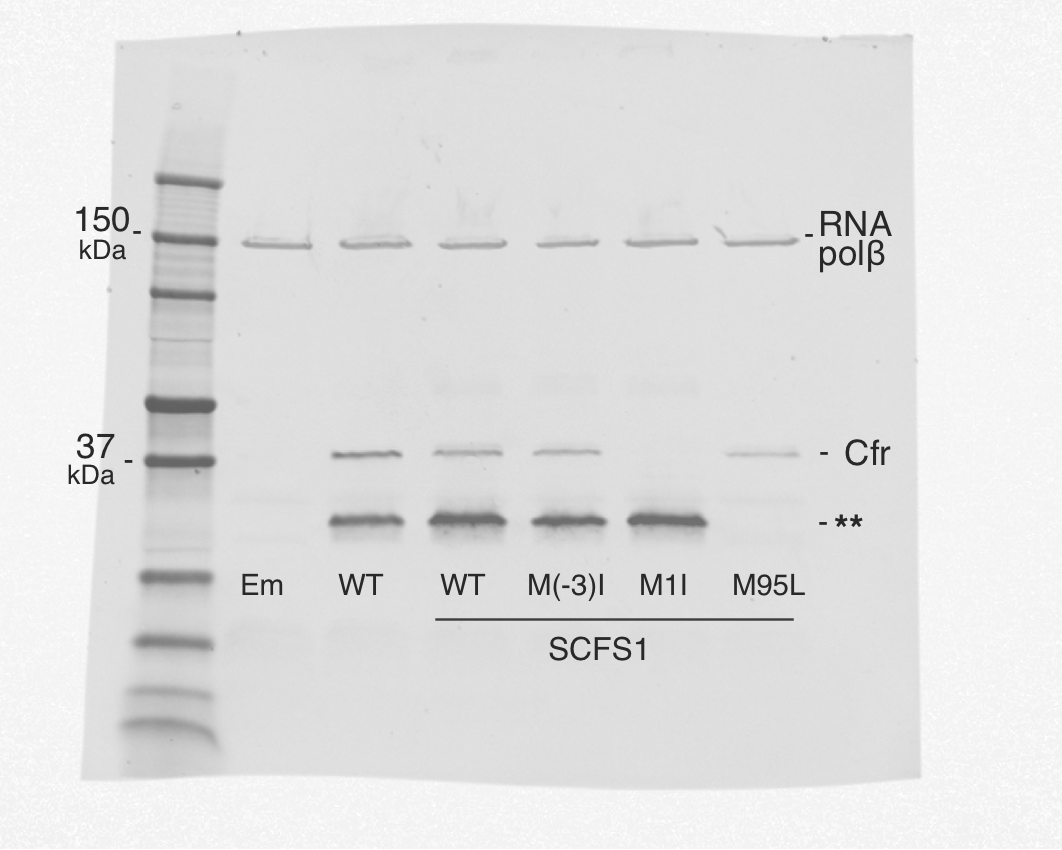

Supplement: Figure 6—source data 2. [file elife-70017-fig6-data2.zip › Figure 6 - Source Data 2 - figure supplement 1/SCFS1 Met Mutants (Labels).tiff]

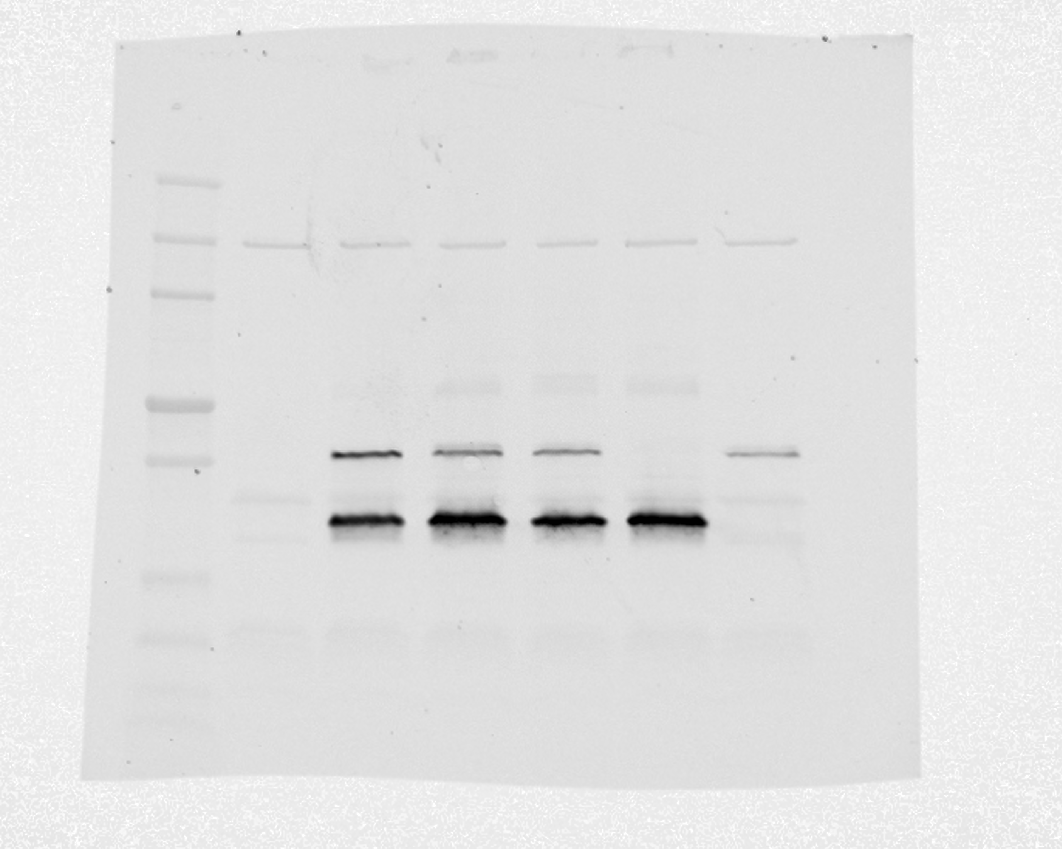

Supplement: Figure 6—source data 2. [file elife-70017-fig6-data2.zip › Figure 6 - Source Data 2 - figure supplement 1/SCFS1 Met Mutants (IRDye 800CW).tif]

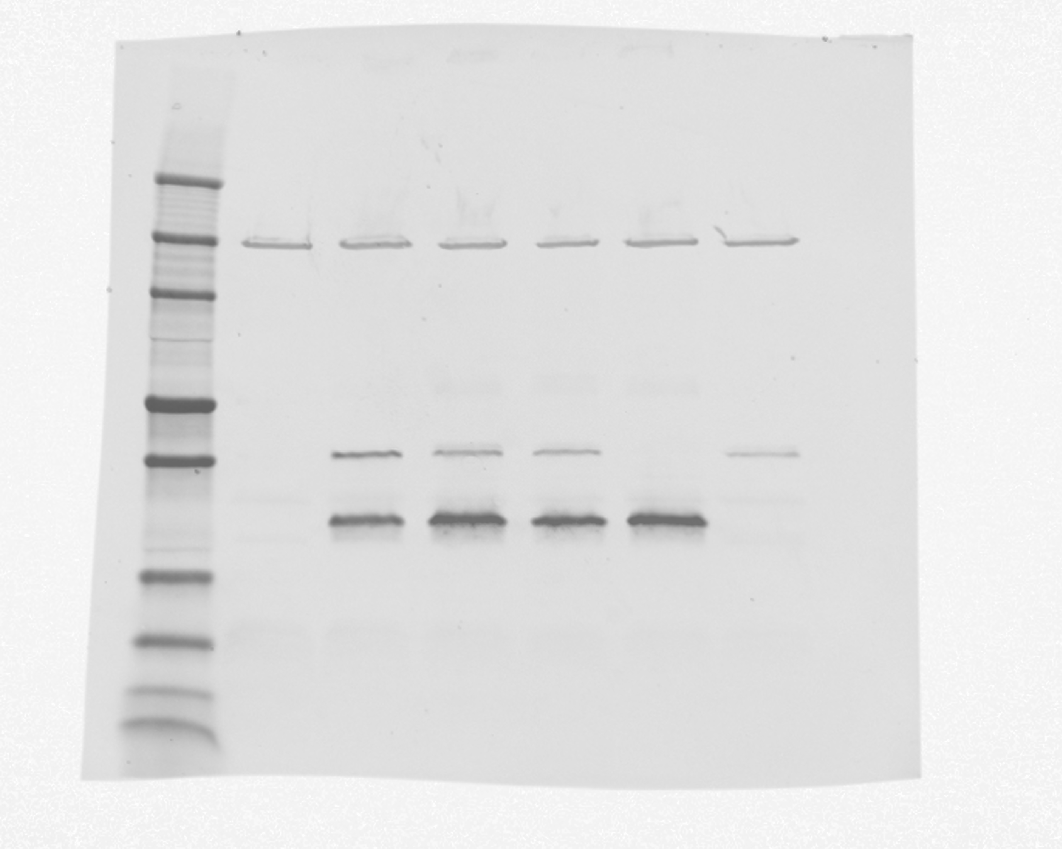

Supplement: Figure 6—source data 2. [file elife-70017-fig6-data2.zip › Figure 6 - Source Data 2 - figure supplement 1/SCFS1 Met Mutants (Composite).tif]

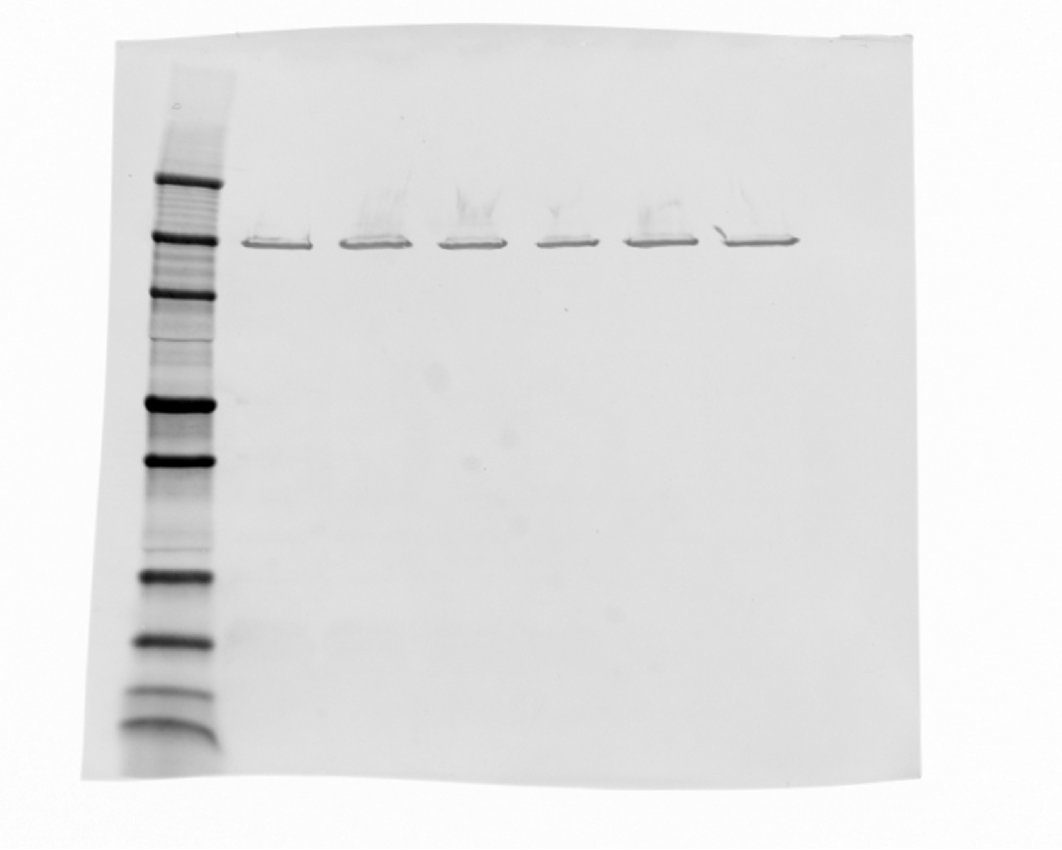

Supplement: Figure 6—source data 2. [file elife-70017-fig6-data2.zip › Figure 6 - Source Data 2 - figure supplement 1/SCFS1 Met Mutants (DyLight 680).tif]
