# Supplementary material for: Directed evolution of the rRNA methylating enzyme Cfr reveals molecular basis of antibiotic resistance
Source: eLife. 2022 Jan 11;11:e70017. doi: 10.7554/eLife.70017 (PMC8752094; doi:10.7554/eLife.70017)
Supplement: Figure 4—source data 1. [file elife-70017-fig4-data1.zip › Figure 4 - Source Data 1/Figure 4 - Source Data 1 Info.docx]

**Original files of the full raw unedited blot (for Figure 4e)**

This zip contains the raw uncropped, unedited blot .tif files for Figure 4e. Files are labeled according to the corresponding channel (IRDye 800CW) and Cfr mutant protein or GAPDH control protein. Labeling of relevant bands can be found in Figure 4 - figure supplement 2.
