# Supplementary material for: Directed evolution of the rRNA methylating enzyme Cfr reveals molecular basis of antibiotic resistance
Source: eLife. 2022 Jan 11;11:e70017. doi: 10.7554/eLife.70017 (PMC8752094; doi:10.7554/eLife.70017)
Supplement: Figure 4—source data 4. [file elife-70017-fig4-data4.zip › Figure 4 - Source Data 4 - figure supplement 2 /Figure 4 - Source Data 4 Info.docx]

**Original files of the full raw unedited blot (for Figure 4 – figure supplement 2a)**

This zip contains the raw uncropped, unedited blot .tif files for Figure 4 – figure supplement 2a. Files are labeled according to the corresponding channel for 2-color detection (IRDye 800CW or DyLight 680) or the composite image. This zip also contains a figure of the uncropped blot with relevant bands for RNA polymerase beta subunit (loading control) and Cfr with its corresponding truncations (asterisks) clearly labeled.
