# Supplementary material for: Directed evolution of the rRNA methylating enzyme Cfr reveals molecular basis of antibiotic resistance
Source: eLife. 2022 Jan 11;11:e70017. doi: 10.7554/eLife.70017 (PMC8752094; doi:10.7554/eLife.70017)
Supplement: Supplementary file 1. [file elife-70017-supp1.pdf]

Supplementary Files for

Directed evolution of the rRNA methylating enzyme Cfr reveals molecular basis of antibiotic resistance

Short title: **Directed evolution of the Cfr resistance enzyme**

Kaitlyn Tsai, Vanja Stojković, Lianet Noda-Garcia, Iris D. Young, Alexander G. Myasnikov, Jordan Kleinman, Ali Palla, Stephen N. Floor, Adam Frost, James S. Fraser, Dan S. Tawfik, Danica Galonić Fujimori

Correspondence to: Danica.Fujimori@ucsf.edu

This file includes:

A. Supplementary files 1A-D

**Primer Name**	**Application**	**Sequence**
GS1-1XFLAG	Fastcloning Insertion	Fwd: 5’-GATTACAAGGATGACGACGATAAGTGAGCGGCCGCAA ACATGGTAC-3’<br>Rev: 5’-CATCCTTGTAATCGCTACCACCACCTTGGCTATTTTGAT AATTACC-3’
CfrN2K(AAA)	Mutagenesis	Fwd: 5’-AGCTAACCGATGAA**A**TTTAATAATAAAAC-3’<br>Rev: 5’-GTTTTATTATTAAA**T**TTCATCGGTTAGCT-3’
CfrN2K(AAG)	Mutagenesis	Fwd: 5’-AGCTAACCGATGAA**G**TTTAATAATAAAAC-3’<br>Rev: 5’-GTTTTATTATTAAA**C**TTCATCGGTTAGCT-3’
CfrN2I(AUU)	Mutagenesis	Fwd: 5’-AGCTAACCGATGA**TT**TTTAATAATAAAAC-3’<br>Rev: 5’-GTTTTATTATTAAA**AA**TCATCGGTTAGCT-3’
CfrN2I(AUA)	Mutagenesis	Fwd: 5’-AGCTAACCGATGA**TA**TTTAATAATAAAAC-3’<br>Rev: 5’-GTTTTATTATTAAA**TA**TCATCGGTTAGCT-3’
CfrI26M	Mutagenesis	Fwd: 5’-TGAGCCTGATTATAGAAT**G**AAACAAATAACCAATGCG-3’<br>Rev: 5’-CGCATTGGTTATTTGTTT**C**ATTCTATAATCAGGCTCA-3’
CfrS39G	Mutagenesis	Fwd: 5’-GATTTTTAAACAAAGAATT**G**GTCGATTTGAGGATATGAA-3’<br>Rev: 5’-TTCATATCCTCAAATCGAC**C**AATTCTTTGTTTAAAAATC-3’
MK(AAA)E-Cfr	Fastcloning Insertion	Fwd: 5’-**ATGAAAGAA**ATGAATTTTAATAATAAAACAAAGTATG GTAAAATACAG-3’<br>Rev: 5’-ATTCATTTCTTTCATCGGTTAGCTTATCGATAC-3’
MK(AAG)E-Cfr	Fastcloning Insertion	Fwd: 5’-**ATGAAGGAAA**TGAATTTTAATAATAAAACAAAGTATG GTAAAATACAG-3’<br>Rev: 5’-ATTCATTTCCTTCATCGGTTAGCTTATCGATAC-3’
CfrM95L	Mutagenesis	Fwd: 5’-GTAGAAACGGTAAAC**C**TGAAGTATAAAGCAG-3’<br>Rev: 5’-CTGCTTTATACTTCA**G**GTTTACCGTTTCTAC-3’
TrunM26-Cfr	Fastcloning Deletion	Fwd: 5’-TCGATAAGCTAACCGATGAAACAAATAACCAATGCG-3’<br>Rev: 5’-CGGTTAGCTTATCGATACCGTCGACC-3’
CfrC338A	Mutagenesis	Fwd: 5’-_ATTGACGCTGCT**GC**TGGTCAATTATATG-3’<br>Rev: 5’-_CATATAATTGACCA**GC**AGCAGCGTCAAT-3’
cfr	RT-qPCR	Fwd: 5’-AGCAGAGCAAAATTCAGAGCAAGT-3’<br>Rev: 5’-TCCAATGTCGCCTGTAGCACAA-3’<br>Length of amplicon: 169 bp
luc<br>Accession no: X65316.2	RT-qPCR	Fwd: 5’-AGATCGTGGATTACGTCGCC-3’<br>Rev: 5’-TGGACTTTCCGCCCTTCTTG-3’<br>Length of amplicon: 156 bp
recA<br>Accession no: CP037857.1	RT-qPCR	Fwd: 5’-ATCGCCTGGCTCATCATACG-3’<br>Rev: 5’-GCACTGGAAATCTGTGACGC-3’<br>Length of amplicon: 152 bp
CfrM(-3)I	Mutagenesis	Fwd: 5’-TTACCACTAGAGCAAATTGTGAAAGGAT**C**AAAGAAATG-3’<br>Rev: 5’-CCTGTATTTTACCATACTTTGTTTTATTATTAAAATTC ATTTCTTT**G**ATCCTTTC-3’
CfrM1I	Mutagenesis	Fwd: 5’-CACTAGAGCAAATTGTGAAAGGATGAAAGAAAT**C**AATT TTAA-3’<br>Rev: 5’-CCTGTATTTTACCATACTTTGTTTTATTATTAAAATT**G** ATTTCTTTCATCCTTTC-3’

Supplementary file 1A. Primer sequences used in this study. All primers were purchased from Integrated DNA Technologies (IDT) or Elim Biopharm and prepared with standard desalting purification methods.

Tiamulin µg/mL	Colony #	Mutations	2nd Codon
400 Enrichment Round 1	2 (CfrV6)	Promoter, I26M, E351Stop, 3'UTR-INS	AAU
	3	**N2K,** S39G, I326V, Q346H, E351Stop	**AAA**
	4	**N2K,** S39G, N57D, S348C, E351Stop, 3'UTR-INS	**AAA**
	5	**N2K**, S18R, E266D, E351Stop	**AAA**
	6 (CfrV2)	**N2K,** S39G, E351Stop	**AAA**
500 Enrichment Round 1	1	**N2K,** I26M, S273R, E351Stop	**AAA**
	2	**N2K,** S39G, N347K, S348Stop	**AAA**
	3 (CfrV3)	**N2K,** I26M, S39G, E351Stop, 3'UTR-INS	**AAA**
	5	**N2K,** S39G, K198N, Q346Stop	**AAA**
	6	**N2K,** S39G, E351Stop, 3'UTR-INS	**AAA**
	7	*N2I*, S39G, Q202H, M301K, E351Stop, 3'UTR-INS	*AUU*
600 Enrichment Round 1	1 (CfrV1)	**N2K,** I26M, E351Stop, 3'UTR-INS	**AAA**
	2	*N2I*, I26M, N73H, E351Stop	*AUU*
	4	**N2K**, N20S, K35R, S39G, L68F, N238D, L265H, E351Stop	**AAA**
	5	*N2I*, S39G, Q349Stop, 3'UTR-INS	*AUU*
700 Enrichment Round 1	1 (CfrV7)	Promoter, S39G, E351Stop, 3'UTR-INS	AAU
	2	N5K, S39G, I233L, E351Stop	AAU
	4	*N2I,* I26M, S39G, G308V, E351Stop	*AUU*
	5	S39G, L68F, G115R, K198R, E351Stop, 3'UTR-INS	AAC*
	6	S39G, L289M, E351Stop, 3'UTR-INS	AAC*
	7	*N2I,* S39G, E351Stop, 3'UTR-INS	*AUU*
	8	**N2K**, I26M, N238D, E351Stop	**AAA**
800 Enrichment Round 1	1	**N2K,** S39G, Q346R, Q349Stop, 3'UTR-INS	**AAA**
	2	*N2I*, S39G, I233L, P259H, Q349Stop, 3'UTR-INS	*AUU*
	4	N5I, K35R, S39G, E351Stop, 3'UTR-INS	AAC*
	5	Promoter, L68F, S348N, E351Stop, 3'UTR-INS	AAU
	6	Promoter, I26M, K45Q, L68F, E351Stop, 3'UTR-INS	AAU
1000 Enrichment Round 2	1	Promoter, *N2I*, D23E, I26M, A305T, Q349Stop, 3'UTR-INS	*AUU*
	2	**N2K,** S39G, Q346R, E351Stop, 3'UTR-INS	**AAA**
	3	**N2K,** I26M, T62A, E351Stop	**AAA**
	4	N5K, S39G, A305T, E351Stop	AAU
	6	**N2K**, S39G, N65S, Q349Stop, 3'UTR-INS	**AAA**
	7 (CfrV5)	*N2I*, S39G, E351Stop, 3'UTR-INS	*AUU*
	8	**N2K,** I26M, Q349Stop, 3'UTR-INS	**AAA**
1250 Enrichment Round 2	2	S39G, L68F, G115R, K198R, E351Stop, 3'UTR-INS	AAC*
	4	Promoter, Y127F, D234G, E351Stop, 3'UTR-INS	AAC*
	5	Promoter, I26M, S39G, Q72K, S85T, E351Stop, 3'UTR-INS	AAU
	6	**N2K,** I26M, N65S, Q349Stop, 3'UTR-INS	**AAA**
	7 (CfrV4)	**N2K,** I26M, L68F, E351Stop, 3'UTR-INS	**AAA**
	8	Promoter, N5K, S39G, S273N, S277R, K315E, E351Stop	AAU
1500 Enrichment Round 2	1	Promoter, **N2K,** R17S, N73H, E351Stop	**AAA**
	2	**N2K**, S39G, S196G, E270K, G308R, K315R, E351Stop, 3'UTR-INS	**AAA**
	4	Promoter, **N2K,** S39G, Q346H, S348I, 3'UTR-INS	**AAA**
	5	Promoter, I26M, Q36L, N347K, S348Stop, 3'UTR-INS	AAU
	6	Promoter, *N2I,* D23E, I26M, A305T, Q349Stop, 3'UTR-INS	*AUU*
	7	**N2K**, S39G, E351Stop	**AAA**
	8	Promoter, S39G, E351Stop, 3'UTR-INS	AAU

Supplementary file 1B. Evolved Cfr sequence variants observed during final enrichment rounds of directed evolution. Open reading frame mutations and alterations to sequences 5' (promoter) and 3' (insertion in 3' untranslated region) of the *cfr* gene are designated. Green lettering designates to the original Asn codon in CfrWT, while green with * designates an Asn synonymous codon. N2K(AAA) codon is in red, while N2I(AUU) codon is in blue.

Enrichment Round	Tiamulin µg/mL	Colony #	Promoter Architecture
-	-	CfrWT	Ptet – *cfr*
1	400	2 (CfrV6)	Ptet – Ins – Ptet – *cfr*
	700	1 (CfrV7)	Ptet – Ins – pPtet – *cfr*
	800	5	Ptet – Ins – pPtet – *cfr*
	800	6	Ptet – Ins – pPtet – *cfr*
2	1000	1	Ptet – Ins – pPtet – *cfr*
	1250	4	Ptet – Ins – pPtet – *cfr*
	1250	5	Ptet – Ins – pPtet – *cfr*
	1250	8	Ptet – Ins – pPtet – *cfr*
	1500	1	Ptet – Ins – pPtet – *cfr*
	1500	4	Ptet – Ins – pPtet – *cfr*
	1500	5	Ptet – Ins – pPtet – Ins – pPtet – *cfr*
	1500	6	Ptet – Ins – pPtet – *cfr*
	1500	8	Ptet – Ins – pPtet – *cfr*

Supplementary file 1C. Promoter architecture of evolved Cfr variants from final enrichment rounds with promoter alterations. Abbreviations: Ptet = promoter; Ins = insertion sequence of various length; pPtet = partial promoter.

pZA	TIA MIC µg/mL
Empty	400-800
CfrWT-GS1-FLAG	1600
CfrV1-GS1-FLAG	6400
CfrV2-GS1-FLAG	6400
CfrV3-GS1-FLAG	3200-6400
CfrV4-GS1-FLAG	6400
CfrV6-GS1-FLAG*	3200
CfrV7-GS1-FLAG*	6400

Supplementary file 1D. Antibiotic susceptibility testing of FLAG constructs. Minimum inhibitory concentration (MIC) required to inhibit bacterial growth of *E. coli* BW25113 transformed with pZA plasmid encoding evolved Cfr variants with a C-terminal glycine-serine linker followed by a FLAG tag. Antibiotic susceptibility testing was performed in two biological replicates by microbroth dilution in the presence of tiamulin (TIA). Asterisk denotes that the 3' insertion sequence after the stop codon (3'UTR), which was introduced during directed evolution, was removed to install the C-terminal tag. Lack of the 3' UTR insertion sequence did not impact resistance for CfrV6/7.
